# In-Depth Molecular Dynamics Study of All Possible Chondroitin Sulfate Disaccharides Reveals Key Insight into Structural Heterogeneity and Dynamism

**DOI:** 10.3390/biom12010077

**Published:** 2022-01-05

**Authors:** Balaji Nagarajan, Nehru Viji Sankaranarayanan, Umesh R. Desai

**Affiliations:** 1 Institute for Structural Biology, Drug Discovery and Development, Virginia Commonwealth University, Richmond, VA 23219, USA; nvsankaranar@vcu.edu; 2 Department of Medicinal Chemistry, Virginia Commonwealth University, Richmond, VA 23298, USA

**Keywords:** chondroitin sulfate, conformational analysis, glycosaminoglycans, hydrogen bonding, molecular dynamics, potential energy surface

## Abstract

GAGs exhibit a high level of conformational and configurational diversity, which remains untapped in terms of the recognition and modulation of proteins. Although GAGs are suggested to bind to more than 800 biologically important proteins, very few therapeutics have been designed or discovered so far. A key challenge is the inability to identify, understand and predict distinct topologies accessed by GAGs, which may help design novel protein-binding GAG sequences. Recent studies on chondroitin sulfate (CS), a key member of the GAG family, pinpointing its role in multiple biological functions led us to study the conformational dynamism of CS building blocks using molecular dynamics (MD). In the present study, we used the all-atom GLYCAM06 force field for the first time to explore the conformational space of all possible CS building blocks. Each of the 16 disaccharides was solvated in a TIP3P water box with an appropriate number of counter ions followed by equilibration and a production run. We analyzed the MD trajectories for torsional space, inter- and intra-molecular H-bonding, bridging water, conformational spread and energy landscapes. An in-house phi and psi probability density analysis showed that 1→3-linked sequences were more flexible than 1→4-linked sequences. More specifically, phi and psi regions for 1→4-linked sequences were held within a narrower range because of intra-molecular H-bonding between the GalNAc O5 atom and GlcA O3 atom, irrespective of sulfation pattern. In contrast, no such intra-molecular interaction arose for 1→3-linked sequences. Further, the stability of 1→4-linked sequences also arose from inter-molecular interactions involving bridged water molecules. The energy landscape for both classes of CS disaccharides demonstrated increased ruggedness as the level of sulfation increased. The results show that CS building blocks present distinct conformational dynamism that offers the high possibility of unique electrostatic surfaces for protein recognition. The fundamental results presented here will support the development of algorithms that help to design longer CS chains for protein recognition.

## 1. Introduction

Sulfated glycosaminoglycans (GAGs) are increasingly being recognized as contributing to biological functions in either their covalently-bound form, as a part of proteoglycans on cell surfaces, or in free solution form, as endogenous polysaccharides or oligosaccharides in biological fluids [1,2,3]. Structurally, multiple features make GAGs unique, including a high level of sulfation, a variable sulfation pattern, and different inter-glycosidic linkages. In addition, GAGs exhibit a large number of conformational states arising from inter-glycosidic bond flexibility as well as occupancy of different saccharide ring puckers [4,5,6]. In combination, GAGs exhibit massive structural and conformational diversity that presumably is the key to their interaction with proteins.

As of 2020, more than 800 biologically important proteins are known to bind to GAGs [7,8]. These proteins contribute to fundamental cellular processes such as growth, differentiation and morphogenesis [9,10,11]. Their roles in the modulation of neuronal functions are particularly intriguing and exciting. On the pathological side, GAGs have been shown to play a role in Alzheimer’s disease, tauopathies, and amyloid diseases [12,13]. A key member of the GAG superfamily is chondroitin sulfate (CS), which is present in the brain and has been associated with neuronal development and dysfunction [14,15,16]. In fact, a specific type of CS, called chondroitin-4,6-disulfate (also called CS-E), has been shown to stimulate neuronal growth and differentiation [16,17]. Likewise, a specific type of CS, called dermatan sulfate (also called CS-B), has been implicated in binding to heparin cofactor II, a human plasma protein involved in inhibiting arterial thrombosis [18,19].

The family of CS polysaccharides has approximately nine members, including CS-A through CS-E and CS-K through CS-M [20], of which CS-B is typically referred to as dermatan sulfate (DS), rather than CS. Of these, CS-A through CS-C are more commonly observed in nature, whereas the remaining are rare biopolymers. The different classes of CS arise due to variations in the sulfation pattern and epimerization state of its building blocks. The two building blocks of CS polysaccharides are *D*-glucuronic acid (GlcA) and *N*-acetyl-*D*-galactosamine (GalNAc). For DS, i.e., CS-B, the former may also be present as *L*-iduronic acid (IdoA). The diversity of information presented by CS polysaccharides is so large that it has remained difficult to harness these molecules for therapeutic purposes. One reason for this is that computational studies on CS in particular, and GAGs in general, are difficult. The large number of building blocks, e.g., 16 in the case of CS, which does not include DS building blocks, imposes significant challenges in terms of computational time and analysis [21,22,23,24]. In fact, no examples of computationally designed CS sequences that have reached a high level of clinical development are available in the literature. Yet, the area of CS sequence design is promising, as indicated by our recent studies, which implicated distinct CS sequences in binding to transforming growth factor beta (TGFβ) [25].

It is important to perform fundamental computational studies on CS building blocks to evaluate their conformational preferences and dynamism in the free solution state. Understanding their structural and energetic preferences may offer better insight into designing oligomeric CS sequences that selectively bind proteins of interest. The literature contains several reports on the atomistic characterization of CS building blocks using X-ray crystallography [26], NMR [27,28,29], molecular modeling (MM) [30] and molecular dynamics (MD) [31,32,33]. Insights from the NMR and crystallographic studies have shown that the backbone orientations of the polymeric scaffold changes as sulfate groups are introduced at various positions. The MM3 force field-based adiabatic mapping of CS disaccharides revealed that the four-sulfated sequences (i.e., CS-A) are more rigid in contrast to non-sulfated or six-sulfated sequences (i.e., CS-C) [34]. Two studies have independently shown the importance of inter- and intra-molecular interactions in stabilizing the secondary structure of CS [31,32]. A recent study concluded that the TIP3P water model is better than four- or five-charged water models in revealing direct intra-molecular hydrogen bonding (H-bonding) in CS [35]. A report based on a biased sampling method studied 10 CS disaccharides in water and counter ions [36] to inform about conformational transitions and energy minima using the CHARMM C36 force field. Another report on 16 CS disaccharides explored their free energy surface using the CHARMM C36 force field and identified conformational dynamism based only torsional angle analysis [37].

Unfortunately, none of these investigations explored conformational dynamism as a function of the nature of inter-glycosidic linkage and the role of water molecules in modulating this dynamism. Additionally, the investigations did not present insights on the possibility of designing CS oligosaccharides that bind to protein targets in a selective manner. We undertook this project with the goal of understanding the conformational dynamism of CS building blocks. We sought to answer the question of whether CS building blocks access distinct conformational states, which may be possible to harness in an oligosaccharide design project. We used an all-atom GLYCAM06 force field to explore the conformational space of all possible CS building blocks. Based on our studies and analysis, including torsional space, inter- and intra-molecular hydrogen bonding (H-bonding), bridging water, conformational spreading (entropy) and energy landscapes, we conclude that CS building blocks present distinct inter-glycosidic bond-based topologies that offer a high possibility of unique electrostatic surfaces for protein recognition. The fundamental results presented here will support the development of algorithms that help in the design of longer CS chains for protein recognition.

## 2. Methods

**Figure 1:**
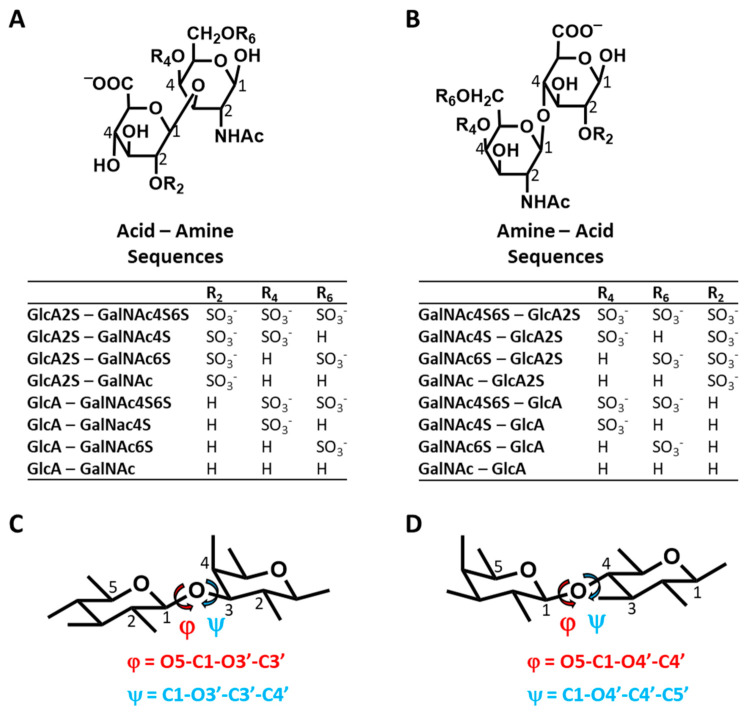
Structures of the two disaccharide classes of CS, i.e., Acid-Amine or β1→3-linked (**A**) and or β1→4-linked Amine-Acid (**B**), studied in this work. Each class consisted of 8 disaccharides with varying sulfation levels, indicated by the R_2_, R_4_ and R_6_ groups. The structures were generated in SYBYL with pre-defined experimental torsion angles marked phi (φ) and psi (ψ) based on structures available in the PDB (protein data bank). The definitions of torsional angles φ and ψ for each disaccharide of the two classes are provided in (**C**,**D**).

**CS Disaccharide Library Construction—**The library of 16 CS disaccharide structures was built and minimized in SYBYLX 1.3 (Tripos Associates, St. Louis, MO, USA). The initial structures had inter-glycosidic angles set to the average of the available crystal structures in the protein data bank (Table S1). Of the 16 disaccharides, eight had β1→3-linked GlcA and GalNAc residues and eight had β1→4-linked GalNAc and GlcA residues with varying numbers of sulfate groups at the 2, 4 and 6 positions (Figure 1). The coordinates of these structures were taken and the atoms and the residue types were changed to GLYCAM06 notations. The molecules were loaded in AMBER18 Xleap (https://ambermd.org) (accessed on 22 November 2021) with GLYCAM06 force field for simulations. Bonds and notations were rigorously checked and the total charge on the molecule was calculated. The charges of sulfate atoms were modified according to the GLYCAM documentation (https://glycam.org) (accessed on 22 November 2021) to ensure a formal charge of −1 for the sulfate group [38].

**Preparations for MD Experiments—**The schematic workflow of the methodology and the analysis are described in Figure S1. Our reviews on the topic also provide additional details for an interested reader [4,39]. Briefly, the total charge of the system was brought to zero by adding the appropriate numbers of the counter ions (Na^+^) (https://ambermd.org/tutorials/advanced/tutorial8/loop4.php) (accessed on 22 November 2021). The sodium cations were not treated any differently in our MD simulations, which implies that the movements of these ions were unconstrained, as would occur in nature.

To mimic the experimental condition, the system was placed at the center of a three-point charged triangulated water molecules (TIP3P) box with minimum distance of 8 Å between the wall and any solute atom. The initial coordinates were generated and saved as corresponding parameter and coordinate files for consecutive processes. The system was relaxed by performing energy minimization in two steps. In the first step, the solute atoms were restrained with a force constant of 500 kcal/(mol·Å^2^) and solvent atoms were relaxed to remove steric hindrance. In the second step, the whole system was minimized without restraint using 2500 iterations of conjugate gradient minimization to achieve a local minimum energy state with a non-bonded cut off of 10 Å.

Following minimization, the system was equilibrated to the required temperature and pressure in three phases with 2 fs integration time steps. The initial velocities were assigned from the Maxwell distribution at the defined temperature using a random number generator. In phase one, the temperature was brought to 300 K using the Berendsen temperature coupling. This was followed by the establishment of a constant pressure using isotopic position scaling in the second phase. All the solute atoms were restrained in the above two phases. In the third phase, the temperature and pressure of the entire system were held constant using NPT ensemble with 2 fs integration time steps. Once the system equilibration setup for NPT was performed and completed, RMSD analysis was performed to ensure that the system was equilibrated well (not shown). This equilibrated system was used to perform production runs. 

**MD Simulations—**Each disaccharide was studied in an identical manner. MD production runs were performed for 20 ns on a VCU cluster (https://chipc.vcu.edu) (accessed on 22 November 2021) of 16 cores. SHAKE constraints were used for all the bonds with the hydrogen atoms throughout the entire process. The trajectory of each MD simulation was recorded every 1 ps, which generated a total 20,000 frames for each disaccharide. Analysis was performed using all 20,000 frames. The tools used to perform these analyses included CPPTRAJ, Visual Molecular Dynamics (VMD), and some in-house scripts in MATLAB and python. RMSD fluctuations during the trajectory were calculated using the rms tag in cpptraj with reference to either the initial structure, the lowest-energy structure or the crystallographic structure. Torsional analysis, performed using VMD, was used to understand backbone movements in the solution. An in-house script based on python was developed for plotting the torsional probability density. Interactions of inter- and intra-molecular H-bond types were calculated using the cpptraj hbond tag. Likewise, bridging water molecules between donor and acceptor atoms of a disaccharide were identified using the cpptraj hbond tag. Conformational subspace analysis was performed for the entire trajectory using in-house MATLAB code. Likewise, principal components analysis (PCA) to view the conformational entropy and potential energy surface (PES) of each disaccharide was carried out using in-house MATLAB code. The in-house scripts will be made available to any researcher interested in using them for their system of interest. 

## 3. Results and Discussion

MD Experimentation and Validation—Although MD simulation of polymeric GAGs is challenging because long, linear and dynamic chains sample a huge conformational space, simulations of oligomeric chains have been performed with relative ease [4,21,40]. Yet, it is important to ascertain that essentially the entire conformational space is sampled within the simulation timeframe. To ensure this, MD experiments on GAG oligosaccharides have been performed over a wide range (ns→µs) [22,23,36,37,41]. It is important to use an appropriate simulation time that ensures optimal sampling without extending the duration unnecessarily. For the smallest oligosaccharides, such as disaccharides, this could be in the nanosecond range. However, it is important to validate the selection. We used MD simulation runs of 20 ns with trajectories being recorded every 1 ps and analyzed various parameters including torsions, hydrogen bonds, water-mediated interactions, and potential energies (Figure S1).

To ascertain that MD experimentation is rigorous, we used four parallel analyses. (1) Three independent simulations were performed for a representative CS disaccharide (GlcA (β1→3) GalNAc6S) using different initial velocities. The trajectories were analyzed for the presence structural nearest neighbors based on the hierarchical agglomerative clustering method, where the minimum distance between clusters is 2 (ε = 2.0), which is recommended for small molecules [42,43]. This affords balanced analysis of clusters and is recommended when the size of clusters is not known. Convergence in clustering is also important to affirm that sampling of the conformational space is good. Figure S2A shows that the three simulations followed a rather identical trend with a small deviation of <10%. (2) The reliability of MD results was also supported by a principal component analysis (PCA) of the three trajectories for the GlcA(β1→3)–GalNAc6S disaccharide. Figure S2B shows the correlation of the first two principal components for each three MD runs. The overlaid data show that each MD run yielded an essentially identical pattern in the PCA subspace, suggesting an equivalent sampling of conformational space. (3) Another established way of validating MD experimentation is to test for convergence with crystal structure geometry. For this, we used the GalNAc4S–GlcA sequence, which is the internal disaccharide of the sequence reported in the protein data bank (ID: 1C4S; Figure S3A) [26]. The comparison of each frame of the trajectory to the geometry in crystalline state showed an excellent convergence of less than 2.28 Å (Figure S3B). Likewise, comparison with the initial and minimum energy geometries showed a deviation of no more than 1.8 Å to 2.2 Å (Figure S3C,D). (4) Finally, we also performed a 400 ns MD simulation for a model disaccharide GlcA–GalNAc6S. This simulation was 20-fold longer than the 20 ns simulations used earlier. Figure S4 shows the phi–psi comparison of the two simulations. Both are essentially identical with minimal changes in the distributions as well as the probability densities (discussed more below). Thus overall, the results indicated that our experimental setup sufficiently sampled the conformational space within the MD timeframe.

**MD of 16 CS Disaccharides and Conformational Heterogeneity—**Using the validated MD experimental setup, we performed simulations of 16 CS disaccharides, which represent all possible building blocks that give rise to all variants of CS polysaccharide, except for CS-B (DS) (Figure 1). As a group, this library represents sequences carrying no sulfates to a maximum of three sulfate groups. The library also enables the study of chain directionality, i.e., GlcA→GalNAc (Figure 1A) or GalNAc→GlcA (Figure 1B). This is not only important because the terminal residues are different but also because the inter-glycosidic linkages are different (β1→3 for Acid–Amine and β1→4 for Amine Acid sequences). Additionally, GlcA2S is a rare modification in polymeric CS and likely enhances the selectivity of interactions, as demonstrated earlier for heparan sulfate [44]. Finally, we studied only the β-variant of the free reducing end substituent because the α-variant is not present in polymeric CS. The definitions of torsional angles used in this study are presented in Figure 1C,D. These are identical to studies reported in the literature, especially for MD works [31,32,36,37].

We first reviewed the range of conformational space sampled by each CS building block using the hierarchical agglomerative clustering method described above. Figure S5 shows the number of distinct clusters displayed during the MD of all 16 disaccharides. To assess whether these distinct conformational structures arise from any changes in ring pucker, we used the BFMP tool in GLYCAM [45]. Figure S6 shows the proportion of the GlcA and GalNAc puckers for all 16 disaccharide pairs over 20 ns. Both residues preferred ^4^C_1_ puckering throughout the trajectory, as expected on the basis of known conformational preferences [46,47]. Thus, the large number of distinct clusters observed for each disaccharide arose from distinct structural geometries that were apart from each other by ε units (=2.0 Å). Overall, irrespective of the directionality of the disaccharides (Acid→Amine or vice versa), the sequences occupied a wide range of conformations and geometries. This was inferred earlier on the basis of first principles but not rigorously documented.

**Figure 2:**
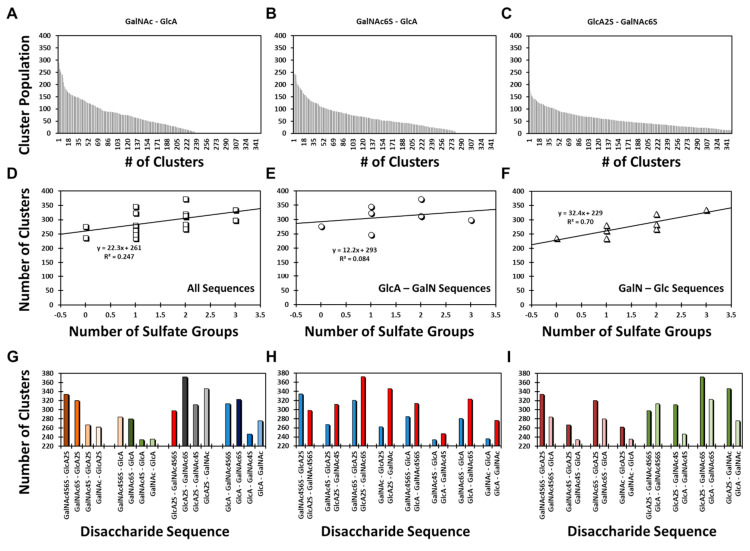
Distinct cluster-based analysis of conformational diversity. Plots of population of clusters as a function of distinct clusters observed in MD simulations of GalNAc–GlcA (**A**), GalNAc6S–GlcA (**B**), and GlcA2S–GalNAc6S (**C**) (see Figure S5 for plots of all 16 disaccharides). Hierarchical agglomerative clustering with the epsilon of 2 was implemented in cpptraj to derive information on distinct clusters observed in MD. Profiles of numbers of distinct clusters observed in MD simulations as a function of the number of sulfate groups on (**D**) all disaccharides, (**E**) 1→3-disaccharides, and (**F**) 1→4-disaccharides. (**G**–**I**) Profiles of the variation in the number of distinct clusters as a function of different sequences.

Yet, it is important to note that the structural diversity was highly varied among different sequences, e.g., a range from ~220 to ~380 clusters. Figure 2A–C present examples of the GalNAc–GlcA, GalNAc6S–GlcA, and GlcA2S–GalNAc6S sequences. The three examples appeared to convey that structural heterogeneity increased with sulfation level; however, the results for all 16 disaccharides revealed a slightly different conclusion. The correlation between structural heterogeneity and number of sulfate groups on a disaccharide was partial at best (R^2^ = 0.247; Figure 2D). When the library was segregated into its two classes, the data revealed that the correlation was non-existent (R^2^ = 0.084) for the GlcA–GalN sequences (Figure 2E), whereas it was fairly strong for (R^2^ = 0.700) for the GalNAc–GlcA sequences (Figure 2F). Because sulfation increases the conformational diversity of GAGs, the result for GalNAc–GlcA sequences was to be expected. Alternatively, the GlcA–GalNAc sequences displayed an abnormal sulfation–structural heterogeneity correlation.

To better compare the structural diversity, we performed three types of analysis including: (1) Comparison of the number of distinct clusters observed for four classes of disaccharides in which GalNAc sulfation increased from 0 to 2. These four classes arose from Acid–Amine (1→3-linkage) and Amine–Acid sequences (1→4-linkage) containing either GlcA2S or GlcA (Figure 2G). For the two Amine–Acid classes, an increase in the number of sulfates on GalNAc resulted in more clusters (i.e., higher structural diversity). In contrast, for the two Acid–Amine classes, this trend was not maintained. (2) Comparison of pairs of Acid–Amine and Amine–Acid sequences carrying an identical number of sulfate groups shows that except for the pair of disaccharides with the most sulfate groups, each Acid–Amine sequence displayed higher structural diversity than the corresponding Amine–Acid disaccharide (Figure 2H). (3) A comparison of the pairs of disaccharides that differed only in GlcA sulfation showed that except for the most sulfated pair, structural diversity was higher for sequences with GlcA2S than for those with GlcA (Figure 2I).

Compilation of these results indicates that the Acid–Amine sequences displayed higher structural diversity that the Amine–Acid sequences, except for the case when sulfation was highest, i.e., GlcA2S–GalNAc4S6S and GalNAc4S6S–GlcA2S. Even here, the anomaly rested with the GlcA2S–GalNAc4S6S sequence, which displayed unusually lower structural diversity (Figure 2G–I). Here, it is instructive to note that whereas the former sequences were found to have 1→3-linked glycosidic bonds, the latter have 1→4-linked glycosidic bonds. This implicates the role of the base inter-residue glycosidic linkage in structural diversity. This base diversity was further enhanced by sulfation, except for the case of GlcA2S–GalNAc4S6S. Although the precise reason for reduced diversity of this sequence remains to be identified, it is possible that the sequence presented reduced degrees of freedom owing to the higher electrostatic repulsion of its four negatively charged groups.

**Torsional Angle Analysis and Structural Diversity—**A systematic conformational analysis of biomolecules relies heavily on torsional angle analysis [48]. We used the standard IUPAC definition of torsional angles, which defines Φ and Ψ as O5-C1-O3′-C3′ and C1-O3′-C3′-C4′ for GlcA–GalNAc sequences, respectively, and O5-C1-O4′-C4′ and C1-O4′-C4′-C5′ for GalNAc–GlcA sequences, respectively. Each frame of the trajectory was analyzed for Φ and Ψ values, which were used to assign structures to unique bins of 3.6° each. The number of structures present in each bin was used to calculate the probability density, which was plotted in a form similar to the Ramachandran plot [48].

**Figure 3:**
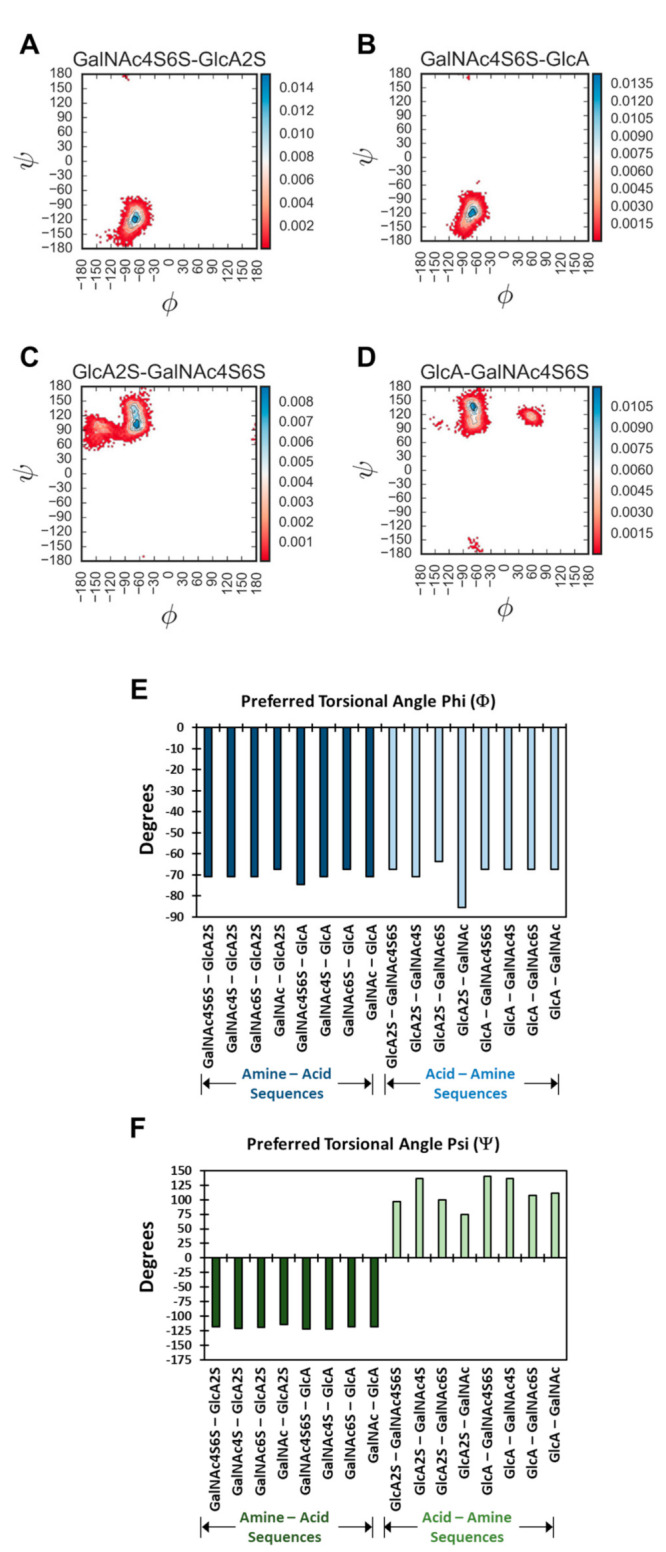
Visualization of torsional populations (phi and psi) within individual bins of identical size (3.6 Å^2^). (**A**–**D**) shows representative disaccharides from the group of 16 (see Figures S7 and S8 for others). The contours shown in the Ramachandran plots refer to probability densities with the blue corresponding to higher probability, while red refers to lower density. (**E**,**F**) present the preferred torsional angles of each disaccharide, which were derived from the high-density regions of corresponding torsional population plots.

Figure 3 and Figure S7 show the probability distribution plots for all 16 disaccharides. Overall, for 1→4-linked sequences, higher probability of occupancy was found to be in the region of −67.2°→−74.5° (Φ) and −114.5°→−121.8° (Ψ). The average spread of Φ and Ψ was found to be 32.5° and 44.7°, respectively, which correlates well with recent studies [36]. For 1→3-linked sequences, these regions were −63.6°→−85.4° (Φ) and +96.3°→+140.0° (Ψ), with an average spread of 21.2° and 61.6° (Φ and Ψ, respectively). These values and ranges are similar to a recent report using the adaptive biased sampling (ABF) simulation, which reported values of 30° and 60° in the presence of sodium [37]. These values are also similar to a recent report using the CHARMM force field [40]. Likewise, these ranges also encompass the torsional preferences displayed by CS oligosaccharides in experimental structures available through the protein data bank (Table S1) [26,27,34,49].

A quick review of probability distributions showed high consistency of profiles for most disaccharides. Yet, interesting variations were evident. For example, the 1→3-linked sequences showed multiple higher probability regions, whereas the 1→4-linked sequences essentially presented a uni-modal distribution (Figure S7). For example, GalNAc4S6S–GlcA and GalNAc4S6S–GlcA2S showed preferences to Φ and Ψ of (−74.5°, −121.8°) and (−70.8°, −118.2°), respectively. In contrast, GlcA–GalNAc4S6S and GlcA2S–GalNAc4S6S showed minimal preferences for (−60.0°, 120.0°) and (−90.0°, 140.0°), respectively (Figure 3) Interestingly, with the 1→3 class of sequences, the sulfation of GlcA (i.e., GlcA2S) induced an additional high-density region (Figure S7). This implies that 1→3-linked sequences containing GlcA2S present distinct conformational heterogeneity.

The probability density plot could be used to identify the preferred torsional angle(s), which corresponded to maximum probability bin(s). As a group, the variation in the preferred Φ and Ψ for the library of 16 disaccharides was not huge, i.e., >±90° (Figure 3E,F and Figure S13). Yet, significant variations were observed for the 1→3-linked sequences, especially in the case of Ψ. 

A question arises regarding the foundational reasons for this structural similarity or variation in Φ and Ψ, especially for the latter. It is well established in the literature that 1,3-diaxial interactions dictate the conformational preferences of the pyranose rings, i.e., ring puckering. In CS, the flexibility of Ψ will likewise be impacted by the bulky groups flanking the torsional angle. For the Acid–Amine (1→3-) sequences, these happened to be axially and equatorially oriented (see Figure 1C), whereas for the Amine–Acid (1→4-) sequences, these were both equatorial (see Figure 1D). In contrast, for Φ, the flanking groups were equatorial and lone pairs of electrons for both the 1→3- and 1→4-linked sequences. This difference could account for the higher flexibility associated with the 1→3-sequences. However, our studies on H-bonding interactions, presented below, indicated another foundational reason for the interesting conformational diversity difference between the two groups of sequences.

**Intra-Molecular Hydrogen Bonds and Conformational Dynamism—**The number and nature of intra-molecular hydrogen bonds (H-bonds) formed during MD simulations were analyzed using cpptraj. H-bonds were defined using 3.0 Å and 135° cut offs between appropriate donor and acceptor atoms, respectively, as described earlier [23,34,50,51]. Figure S9 shows the formation of H-bonds, if any, in conformations corresponding to the global minimum for all 16 disaccharides. As a group, only a limited number of intra-molecular H-bonds were observed, suggesting that the global minima tend to exhibit open topologies.

**Figure 4:**
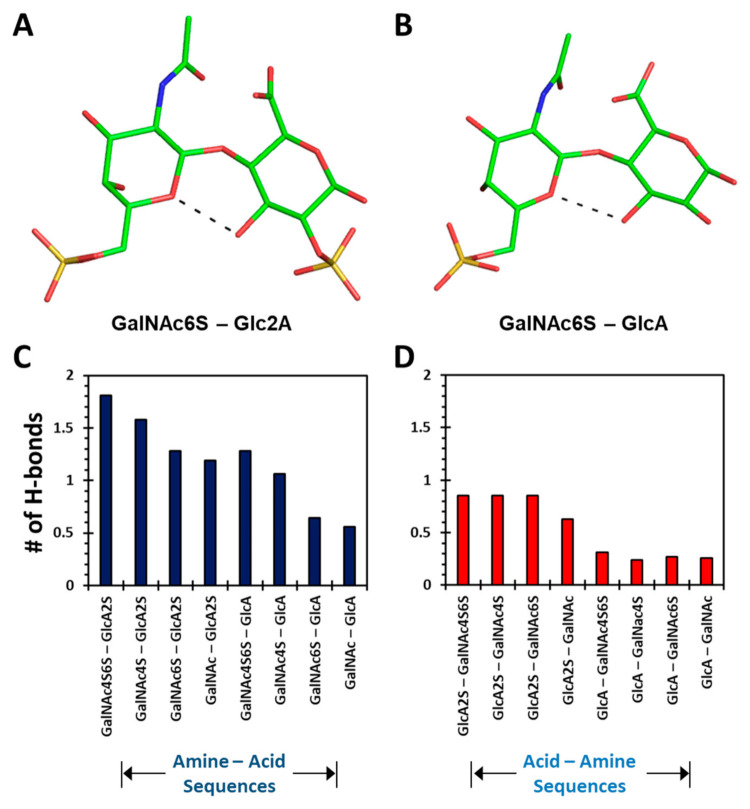
Differences in intra-molecular hydrogen bonds observed during MD simulations of different 1→4- and 1→3-linked sequences (hydrogens are not shown to improve clarity). (**A**,**B**) show intra-molecular H-bonds formed for two representative disaccharides, GalNAc6S–GlcA2S and GalNAc6S–GlcA, respectively. (**C**,**D**) show the average number of intra-molecular H-bonds calculated from the 20,000 frames of each MD trajectory of 8 CS disaccharides of either Amine–Acid (1→4) or Acid–Amine (1→3) type, respectively. Whereas intra-molecular H-bonds are formed between atoms of the CS disaccharide, inter-molecular H-bonds are formed with solvent water molecules. The criteria for defining a H-bond is described in the Methods section.

Yet, interesting differences were observed when considering intra-residue H-bonds formed in the 1→3- and 1→4-linked disaccharides. Only two 1→4-linked sequences displayed intra-residue H-bonds (Figure 4A,B). Both these sequences presented a H-bond between the O5 of GalNAc6S and O3 atoms of GalNAc6S and GlcA, respectively. These bonds have been observed in two earlier studies of CS oligosaccharides using adiabatic mapping with the MM3 force field [34] and a biased sampling method with the CHARMM force field [36]. As is evident from Figure S9 (Panels I→P), the global minima of the 1→3-linked disaccharides did not display any inter-residue H-bond. 

Outside the global minimum of each disaccharide, inter-residue H-bonds arose in many other conformational states. Table S2 and Table S3 list these for GalNAc–GlcA (1→4-linkage) and GlcA–GalNAc (1→3-linkage), respectively. Both tables show that all inter-residue H-bonds were transient in nature and none existed 100% of the time. Several H-bonds of 1→4-linked disaccharides were fairly stable and existed in 20–50% of the MD frames (Table S2). In contrast, the inter-residue H-bonds of 1→3-linked sequences existed no more than 8.5% of the time (Table S3). 

Figure S10 shows the occurrence of the number of intra-molecular H-bonds as a function of MD simulation time for each disaccharide sequence. The data reveal that at any given time, more intra-molecular H-bonds were observed for 1→4-linked disaccharides (Panels A→H) than for 1→3-linked sequences (Panels I→P). This represents a fundamental difference between the two types of sequences. In fact, the average number of H-bonds increased with level of sulfation for the two classes of disaccharides. For the GalNAc–GlcA (1→4-linked) class, the intra-molecular H-bonds increased from 0.56 to 1.81 as the level of sulfation changed from 0 to 4 (Figure 4C). In contrast, the GlcA–GalNAc (1→3-linked) series presented an increase from 0.26 to 0.85 for an identical change in sulfation (Figure 4D).

These results indicated that the intra-molecular H-bonds were highly dynamic for both the 1→4- and 1→3-linked sequences. Conformational dynamism was supported because very few H-bonds were consistently formed across the entire MD trajectory. Within the two classes, the 1→3-linked sequences exhibited much lower numbers of intra-molecular H-bonds as compared to the 1→4-linked sequences. The results further confirmed the fundamental difference between the 1→4- and 1→3-linked topologies.

**Inter-Molecular H-Bonds and Topological Preferences—**Although sulfate groups dominate the interaction of GAGs with proteins, solvent molecules, i.e., H_2_O, play a key role in generating and maintaining local topologies for protein recognition [4,23,39,52]. In fact, nearly all crystal structures display GAG-bound water molecules [52]. Thus, to understand the nature and role of water molecules in the conformational dynamism of CS disaccharides, we analyzed the MD trajectories of inter-molecular H-bond formation, in a manner that was similar to the approach used for those of intra-molecular H-bonds, using cpptraj. Here, the donor and acceptor atoms were from either the solvent (i.e., water) or the solute (i.e., CS atoms). The average numbers of water molecules involved in H-bonding per CS disaccharide are shown in Figure S11. This number ranged from 16 for disaccharides with no sulfates to 28 for sequences with three sulfate groups. Of these, each sulfate group interacted with 5 to 6 water molecules, irrespective of the location of sulfates (Figure S11B). In contrast, the non-sulfated atoms of a disaccharide bound to 11 to 16 water molecules, which equated to approximately 1 to 2 water molecules per OH group (Figure S11C). Interestingly, the directionality of the sequence did not influence the inter-molecular H-bonding. These features imply that CS disaccharides are highly solvent bonded.

Water molecules are also known to bridge two ring atoms within GAG chains. Such bridged water molecules play critical roles in maintaining the conformational preferences of ring puckers as well as torsional angles [23]. Figure S12 shows examples of such water molecules for each of the CS disaccharides. Although transient, bridged water molecules were found in >20% of MD frames, suggesting their role in stabilizing the respective topologies, a common feature observed 1→3-linked sequences was that the sulfation of GlcA added a bridged water molecule interaction (Figure S12M–P), which was less likely for corresponding 1→4-linked sequences (e.g., Figure S12E–H). Likewise, differences in the number of bridged water molecules also arose between the two series of disaccharides (1→3- *v/s* 1→4-) when devoid of sulfation (see Figure S12H *v/s* Figure S12P).

**Figure 5:**
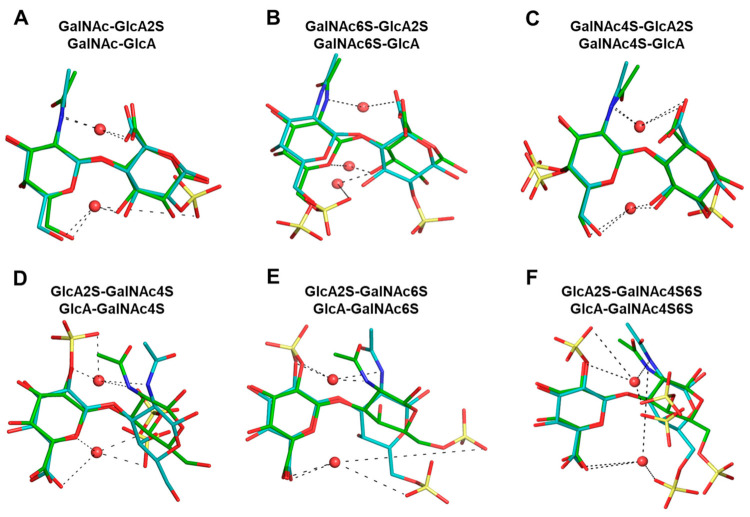
Comparison of topologies of representative sequences from 1→4- (**A**–**C**) and 1→3-linked (**D**–**F**) sequences**.** The backbones (nuclear atoms only) of the non-reducing end residues of selected disaccharides were aligned to evaluate the role of water-mediated interactions in topological preferences. Higher and lower sulfated sequences are shown in cyan and green sticks, respectively. Bridging water molecules are shown as red spheres. See Figures S13 and S14 for further details.

**The Role of Inter- and Intra- Molecular Hydrogen Bonds—**As stated above, the formation and breakdown of H-bonds contribute to the dynamism of conformational topologies. Yet, to pinpoint the role of H-bonds in the modulation of CS topology more clearly, we overlaid representative structures from the torsional probability density plots shown in Figure S7. Figure 5 shows these overlaid plots for selected 1→3- and 1→4-linked sequences. At a very fundamental level, although water-mediated interactions were observed for both sequence classes, their contributions were different. Water-mediated interactions tended to arise from sulfate groups for the 1→3-sequences, whereas for the 1→4-sequences these originated from nuclear atoms. This resulted in higher topological diversity for the 1→3-sequences. More specifically, the presence or absence of two-sulfate in the GlcA of 1→3-sequences led to a variation of ~40% in the bridging water molecules for GalNAc6S (Figure 5E). This percentage dropped dramatically to 4% for GalNAc4S-containing 1→3-sequences (Figure 5D). This implies that protein residues may be able to displace bridging water molecules to engineer a tight fit, as was previously shown for chondroitinase B complexed to CS-A tetrasaccharide (see PDB ID:1OFM) [53].

Figures S13 and S14 show the overlaid structures for 1→3- and 1→4-linked sequences, respectively, which further pinpoint differences within each of these classes. When a sulfate was present in the GlcA residue, each 1→3-linked sequence tended to prefer a more linearized topology because of the higher levels of H-bonding (Figure S13E–H). For non-sulfated GlcA residues this topology is less favored (Figure S13A–D). Likewise, four-sulfate in GalNAc tended to reduce torsional spread because of H-bonding with GlcA residues. This appears to be the reason behind the rigidity of four-sulfated CS sequences, a conclusion supported by earlier observations with the MM3 force field [34]. 

For the 1→4-linked sequences, Figure S7 (above) shows an essentially single and tight probability density. When different topologies from the probability density plots are compared, the overlays not only clarify that the changes are minimal but point to the role of water-mediated interactions (Figure S14). A rather common H-bond between the O5 atom of GalNAc and the O3 atom of GlcA occurred for all disaccharides (see also Table S2), which stabilized each sequence in an essentially identical manner. Likewise, a water-mediated H-bond also arose between the amide of GalNAc and the carboxylate of GlcA. In fact, these H-bonds were found to exist essentially over the entire MD timeframe, resulting in a well-defined topology.

Overall, this analysis provides a foundation for the rather similar topologies, irrespective of the sulfation pattern, observed for the 1→4-linked sequences. Sulfation tended to influence the 1→3 torsional space more than the 1→4 torsional space because of underlying differences in water-mediated interactions.

**Energy Landscape and Multiplicity of Local Minima—**Although most studies focus on the global minima and its role in protein recognition, it is important to recognize that proteins may recognize less populated topologies. The potential energy surface (PES) offers an overview of the different topologies sampled by molecules [23,54]. We utilized MD trajectories to elucidate the PES of all 16 CS disaccharides. PES can be visualized in many ways by plotting two different molecular components (e.g., atom positions, bond angles, etc.) as *x-* and *y*- components against the energy of the system as the *z*-component. For this, each MD frame was quenched using the steepest descent minimization protocol followed by conjugate gradient minimization (maximum 1000 iterations) and then analyzed for potential energy [55]. Simultaneously, we performed principal component analysis (PCA) on all MD frames to derive the first two principal components, which, when plotted against the energy, gave the two-dimensional potential energy landscapes shown in Figures S15 and S16.

**Figure 6:**
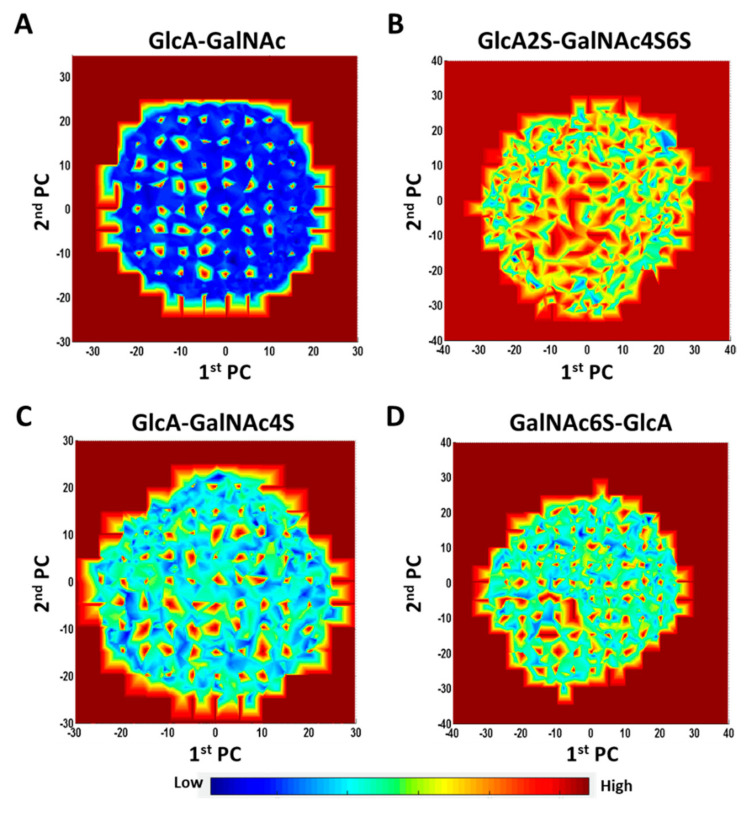
Inherent potential energy landscape of CS disaccharides. Representative landscapes are shown for GlcA-GalNAc (**A**), GlcA2S-GalNAc4S6S (**B**), GlcA-GalNAc4S (**C**) and GalNAc6S-GlcA (**D**). The 2D-contour plots were prepared by projecting the respective potential energy values of the Z axis to the X and Y axes, where the X and Y-axes are the first two principal components (PCs) derived from principal component analysis (PCA) of the MD trajectory representing the conformational subspace. (see Figures S15 and S16 for other disaccharides).

Both the 1→4- and 1→3-linked disaccharides displayed energy landscapes with multiple low- and high-energy coordinates (blue and red *loci*, respectively, in Figures S15 and S16). This implies the existence of kinetic traps, saddle points and local minima. Yet, the energy landscapes of different sequences were significantly different as shown in Figure 6. Unsulfated 1→3-linked disaccharide (GlcA–GalNAc) showed a primarily two-state landscape (high and low energy) (Figure 6A). Likewise, maximally sulfated 1→3-linked disaccharide (GlcA2S–GalNAc4S6S) also showed a two-state landscape, except that saddle points replaced the low-energy states (Figure 6B). In contrast, the intermediate sulfated sequences, e.g., GlcA–GalNAc4S (Figure 6C) or GalNAc6S–GlcA (Figure 6D), presented a rugged energy landscape containing multiple energy states. Considering that fewer CS sequences in nature are either maximally sulfated or non-sulfated, the multiplicity of local minima would dominate the conformer population in nature.

We also compared principal components for selected disaccharides to understand changes in topological spread as a function of sulfation. For glycans, the dynamical motion across an MD trajectory can be captured using multiple parameters such as the root-mean-square deviation (RMSD), end-to-end distance (EED), minimum volume enclosing ellipsoid (MVEE), radius of gyration (RGYR), potential energy (PE), etc. Understanding these changes in toto is difficult because the dynamical profile is multi-dimensional. Principal component analysis (PCA) is an effective decomposition approach that reduces the multi-dimensional space to two dimensions, which capture the most important parameters in a composite form. The PCA for the 16 CS disaccharides clearly revealed similarities and differences among them. The comparison of PCA plots for GlcA2S–GalNAc4S6S and GlcA–GalNAc clearly shows that sulfation enhanced the occupancy of more topologies (Figure S17A). Likewise, the location of sulfates also influenced the conformational spread, as exemplified by the 1→4-linked sequences carrying GalNAc6S or GalNAc4S (Figure S17B,E).

## 4. Conclusions

GAGs are known to bind to numerous proteins and modulate their functions [7,8]. A common explanation for these roles is their electrostatic interactions with positively charged domains on proteins. Electrostatic interactions are typically non-selective, and are difficult to employ in the discovery of drug-like candidates. This is one of the main challenges in translating GAG sequences into clinical candidates. Despite this, selective GAG–protein systems have been found, including heparin–antithrombin [56], heparan sulfate hexasaccharide–heparin cofactor II [57], heparan sulfate octasaccharide–glycoprotein D [58], dermatan sulfate hexasaccharide–heparin cofactor II [18], etc. 

CS has been found to have major roles in neurobiology [9,12,13,14]. Although oligosaccharide sequences with high selectivity for target proteins have yet to be identified, CS-E (GalNAc4S6S) has been found to be a powerful neuronal modulator [2,15,16,17]. Likewise, our recent computational work identified dual site interactions between TGFβ and distinct CS oligosaccharides [25]. These results imply that selective CS sequences should be possible to design and/or discover. Unfortunately, little is known about the structural and conformational properties of the full range of CS building blocks. Thus, this work was directed towards understanding the conformational diversity and dynamism of CS building blocks so as to inform future CS design algorithms.

This is the first study of all possible CS disaccharides using the GLYCAM06 force field. We utilized essential MD on an NPT ensemble to explore the conformational space of 16 disaccharides including GalNAc4S6S and GlcA2S, which are relatively less populated in nature. We first validated our 20 ns MD protocol using one disaccharide sequence. Application of this protocol to all 16 disaccharides revealed some key insights into the structure and dynamics of these key building blocks. It is important to note that 20 ns MD simulations for disaccharides, as performed in this work, were sufficiently robust to offer conformational and dynamic inferences. We expect that longer GAG sequences would require longer simulation times, e.g., 1000 ns, followed by a validation exercise, such as that performed in this work.

The Ramachandran plots showed characteristic preferences for both the 1→4- and 1→3-linked glycosidic bonds, which matched the available crystallographic data of free and protein-bound oligosaccharides (Table S1). These results also correlated well with the published data using MD and NMR [27,31,32,33,36,37]. However, the collective analysis of all possible sequences revealed clear topological differences between the 1→4- and 1→3-linked sequences, each of which exhibited variations arising from the level of sulfation in two saccharide rings. In fact, the 1→3-linked disaccharides showed distinct multiple minima, whereas the 1→4-linked disaccharides presented a uni-modal torsional distribution. Further, sulfation tended to influence the 1→3 conformational space more than that of 1→4 because of the reduced level of inter-residue H-bonding in the former. Individual sulfate groups also influenced conformational diversity. GalNAc6S introduced higher torsional spread than GalNAc4S. Likewise, GlcA2S induced higher conformational diversity than its non-sulfated counterpart. 

The above conclusions, derived from torsional analysis, were also supported in an independent analysis of distinct clusters formed during MD simulations. Here, the 1→3-disaccharides displayed higher structural diversity than the 1→4-sequences. However, an anomaly was observed with the GlcA2S–GalNAc4S6S sequence, which displayed reduced diversity as compared to its 1→4 counterpart (Figure 2). We predict that high electrostatic repulsion from nearest neighbors (three sulfates and one carboxylate) combined with the 1→3-glycosidic bond reduced the number of degrees of freedom.

The results from the intra-molecular H-bonds also differentiate the 1→4- and 1→3-linked sequences. However, both classes of disaccharides were found to be conformationally dynamic because of the presence of relatively few intra-molecular H-bonds (Figure 4). Similar conclusions could be derived from the study of bridging water molecules (Figure 5), which contributed to the stabilization of distinct topologies. In fact, sulfation engineered a higher level of topological diversity for the 1→3-sequences because of a huge variation in the proportion of bridging water molecules in different sequences. However, GlcA2S induced an additional high-density torsional region that presented a distinct topology. For the 1→4-linked sequences, a common water-mediated interaction between the O5 atom of GalNAc and the O3 atom of GlcA was observed for each sequence, irrespective of sulfation, which was important for the stability of distinct topologies. A corollary of these conclusions is that such principles could be employed in the design of a CS oligosaccharide of a defined topology.

Finally, our studies on the quenching of MD frames followed by PCA of conformational space yielded more evidence of a large number of metastable states for all sulfated disaccharides (Figure 6). On the structural extremes, GlcA–GalNAc and GlcA2S–GalNAc4S6S showed the widest difference in topological space. However, subtle variations in conformational spread were also evident for sequences carrying sulfates at different positions, e.g., GalNAc6S *v/s* GalNAc4S.

Overall, our studies indicated the accessibility of distinct topologies by the 1→4- and 1→3- linked disaccharides. Class members exhibited similar, but not identical, preferred geometries. Modulations in these arose due to the presence of sulfate groups at various positions. The six-sulfate group afforded significantly more conformational dynamism than the four-sulfate group. In contrast, the 4,6-disulfate group was likely to present reduced conformational dynamism, especially for the 1→3-linked disaccharide. These lucid conclusions should help design a CS sequence that is selective for its target. An algorithm could be developed based on these studies to design sequences with either fairly linear or bent CS oligosaccharide with a defined number of sulfate groups decorating the topological shape. Likewise, it may also be possible to design a CS sequence with appropriate rigidity or flexibility to occupy a site of binding on the target protein.

## Data Availability

Data and probability density scripts are available upon request.

## Supplementary Materials

The following supporting information can be downloaded at: https://www.mdpi.com/article/10.3390/biom12010077/s1, The accompanying supplementary information contains Figures S1–S17 and Tables S1–S3, which offer additional details on the data and results presented in the main manuscript.
