# Supplementary material for: In-Depth Molecular Dynamics Study of All Possible Chondroitin Sulfate Disaccharides Reveals Key Insight into Structural Heterogeneity and Dynamism
Source: Biomolecules. 2022 Jan 5;12(1):77. doi: 10.3390/biom12010077 (PMC8773825; doi:10.3390/biom12010077)
Supplement: Supplementary file 1 [file biomolecules-12-00077-s001.zip › biomolecules-1502082-supplementary.pdf]

Supplementary Information

for

In-Depth Molecular Dynamics Study of All Possible Chondroitin Sulfate Disaccharides Reveal Key Insight into Structural Heterogeneity and Dynamism

Balaji Nagarajan,[1,2]*, Nehru Viji Sankaranarayanan[1,2] and Umesh R. Desai[1,2]*

[1]*Institute for Structural Biology, Drug Discovery and Development*

and

[2]*Department of Medicinal Chemistry, Virginia Commonwealth University, Richmond, VA 23298, USA*

*Addresses for correspondence: Drs. Balaji Nagarajan (bnagarajan@vcu.edu) and Umesh R Desai (urdesai@vcu.edu), 800 E. Leigh Street, Suite 205, Richmond, VA 23219. Ph (804) 828-7575; Fax (804) 827-3664

#	Table of Contents	Pg.
1	**Figure S1.** MD protocol used for 16 CS disaccharides.	S2
2	**Figure S2.** Test 1 for convergence of sampling.	S3
3	**Figure S3.** Test 2 for convergence of sampling.	S4
4	**Figure S4.** Comparison of 20 ns and 400 ns torsional density.	S5
5	**Figure S5.** Plot of population of clusters as a function of distinct clusters.	S6
6	**Figure S6.** Proportion of ring puckers for each monomer of disaccharides.	S7
7	**Figure S7.** Torsional density distributions across phi and psi.	S8
8	**Figure S8.** Average phi and psi values from MD simulations.	S9
9	**Table S1.** Torsional angles from the available experimental structures.	S10
10	**Figure S9.** Global energy minima observed in MD simulations.	S11
11	**Figure S10.** Number of intra-molecular hydrogen bonds observed in MD frames	S12
12	**Figure S11.** Number of inter-molecular H-bonds observed in MD simulations.	S13
13	**Table S2.** H-bond donors and acceptors for the 1→4-linked disaccharides.	S14
14	**Table S3.** H-bond donors and acceptors for the 1→3-linked disaccharides.	S15
15	**Figure S12.** Inter-residue bridging water molecules observed in MD simulations.	S16
16	**Figure S13.** Overlay of representative topologies for 1→4-linked sequences.	S17
17	**Figure S14.** Overlay of representative topologies for 1→3-linked sequences.	S18
18	**Figure S15.** Energy landscape of the eight 1→4-linked disaccharides.	S19
19	**Figure S16.** Energy landscape of the eight 1→3-linked disaccharides.	S20
20	**Figure S17.** Comparisons of two principal components from PCA of MD trajectories.	S21

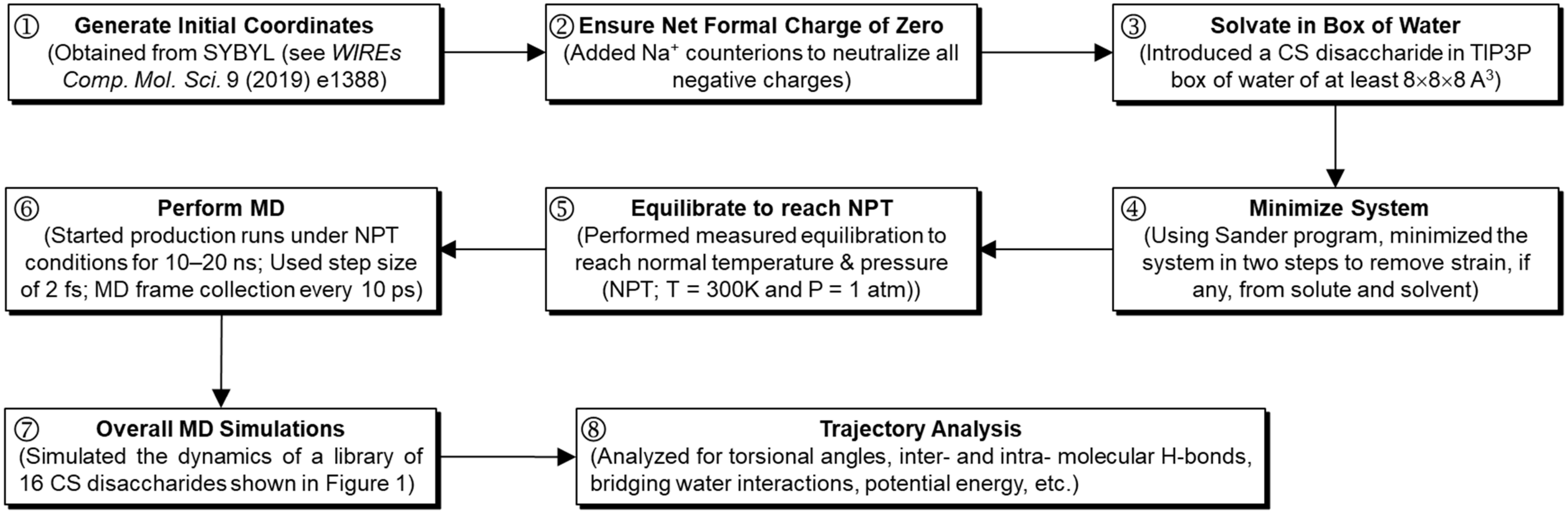

Figure S1. Experimental flow of steps used to perform the MD simulation of each CS disaccharide using GLYCAM06 force field and the AMBER simulation package.

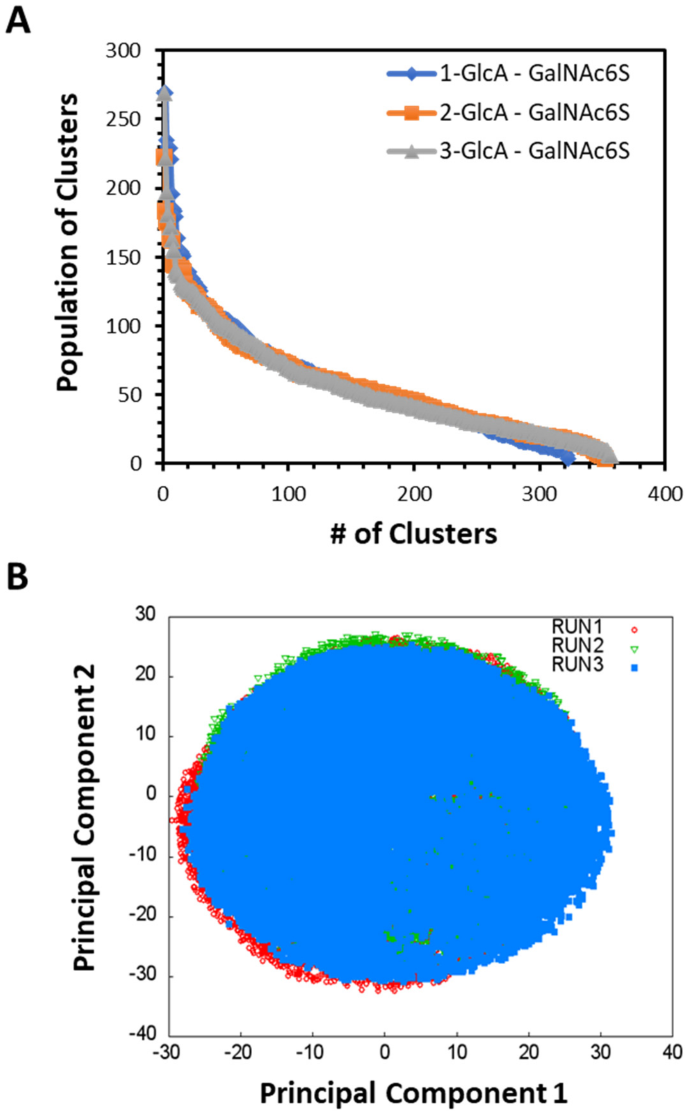

Figure S2. First test for assessing convergence of sampling. A) Shows the clustering of GlcA–GalNAc6S sequence in three different MD simulations. B) Shows joint projection of the principal component analysis from the three independent MD runs. The three MD runs were performed using different initial velocity distributions to assess possible variations in conformational space.

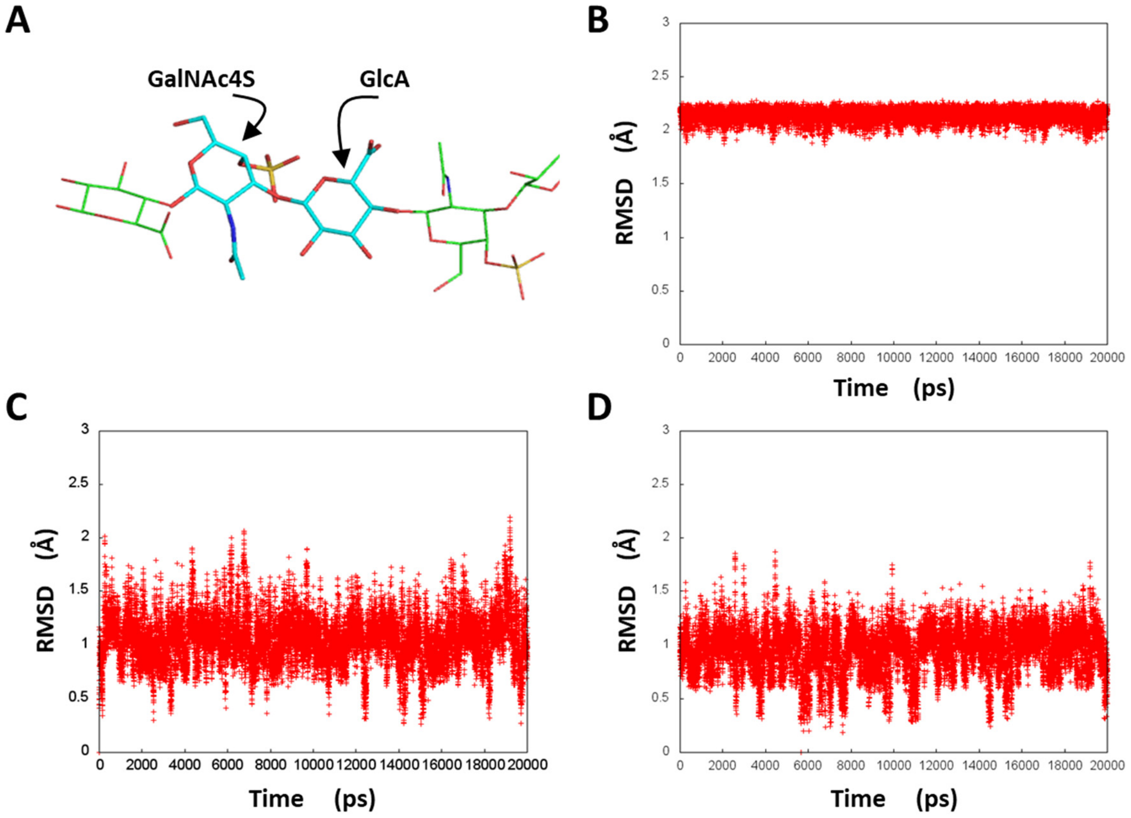

Figure S3. Test 2 for convergence of sampling. A) Crystal structure of a CS tetrasaccharide from the protein data bank (1C4S.pdb). The central disaccharide in this structure is GalNAc4S–GlcA. B) Comparison of the crystal structure to the MD trajectory of GalNAc4S–GlcA. The RMSD fluctuated less than 2.3 Å validating the MD protocol used in the study. C) Likewise, the RMSD between the MD frames and the initial starting structure optimized using SYBYL showed fluctuations within 1.8 Å. D) Similarly, comparison with the lowest energy structure obtained from MD with each of the MD frames showed fluctuations within 2.0 Å.

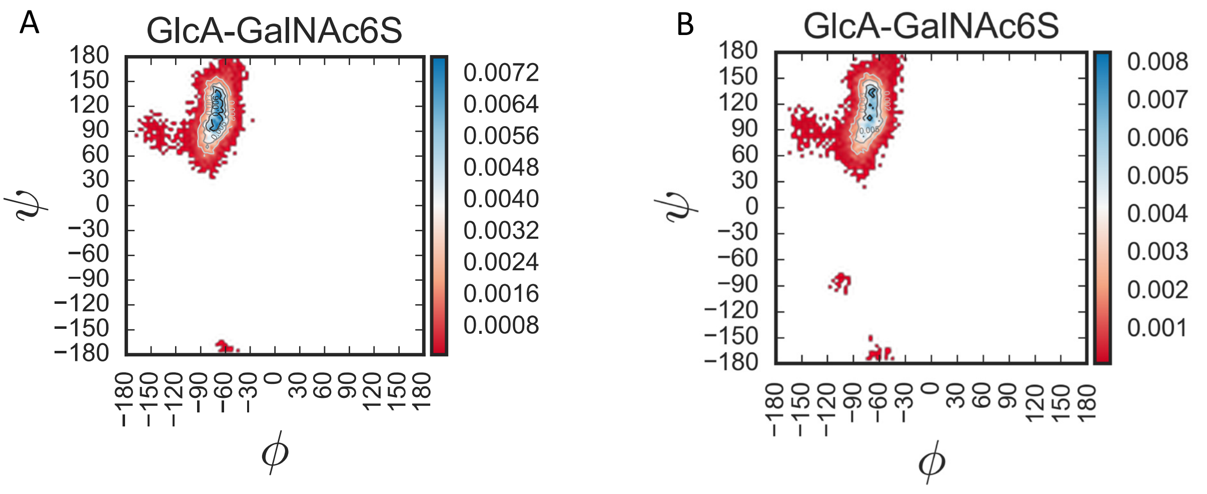

Figure S4. Comparisons of A) 20ns and B) 400ns MD trajectory of CS GlcA-GalNAc6S. Phi-psi density profiles are shown. The density map shows insignificant difference between the two experiments. The simulation also showed similar occurrence of puckering percentage for GlcA and GalNAc6S (not shown).

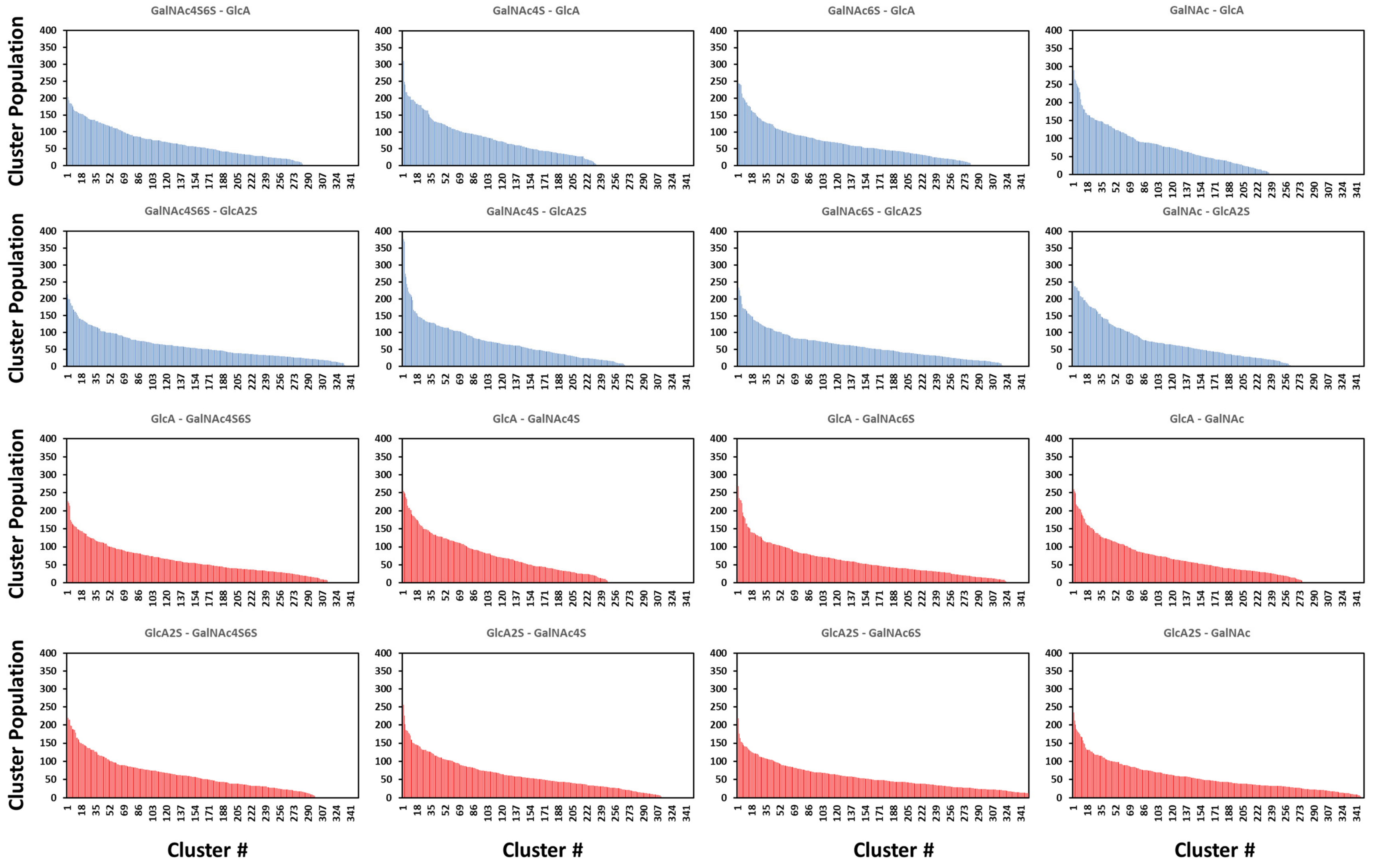

Figure S5. Plot of population of clusters as a function of distinct clusters. Profiles for all 16 CS disaccharide studied here are shown. Hierarchical agglomerative clustering with the epsilon of 2 was implemented in cpptraj.

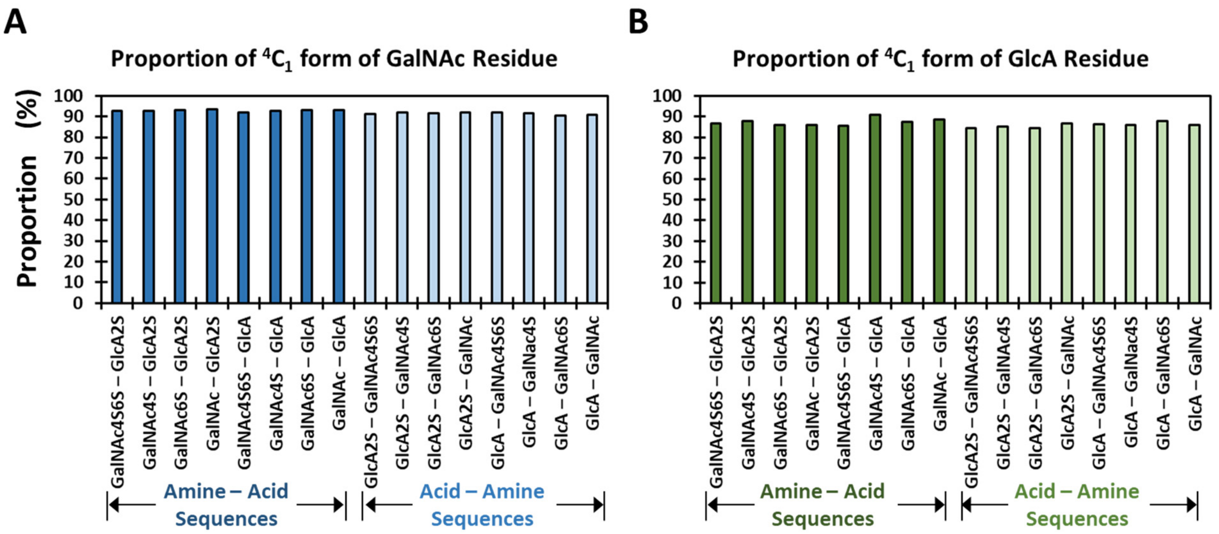

Figure S6. Proportion of $^4C_1$ puckers of GalNAc (A) and GlcA (B) residues constituting the 16 CS disaccharides of either GalNAc–GlcA (i.e., Amine–Acid) or GlcA–GlcNAc type (i.e., Acid–Amine). No other ring puckers were observed for any residues.

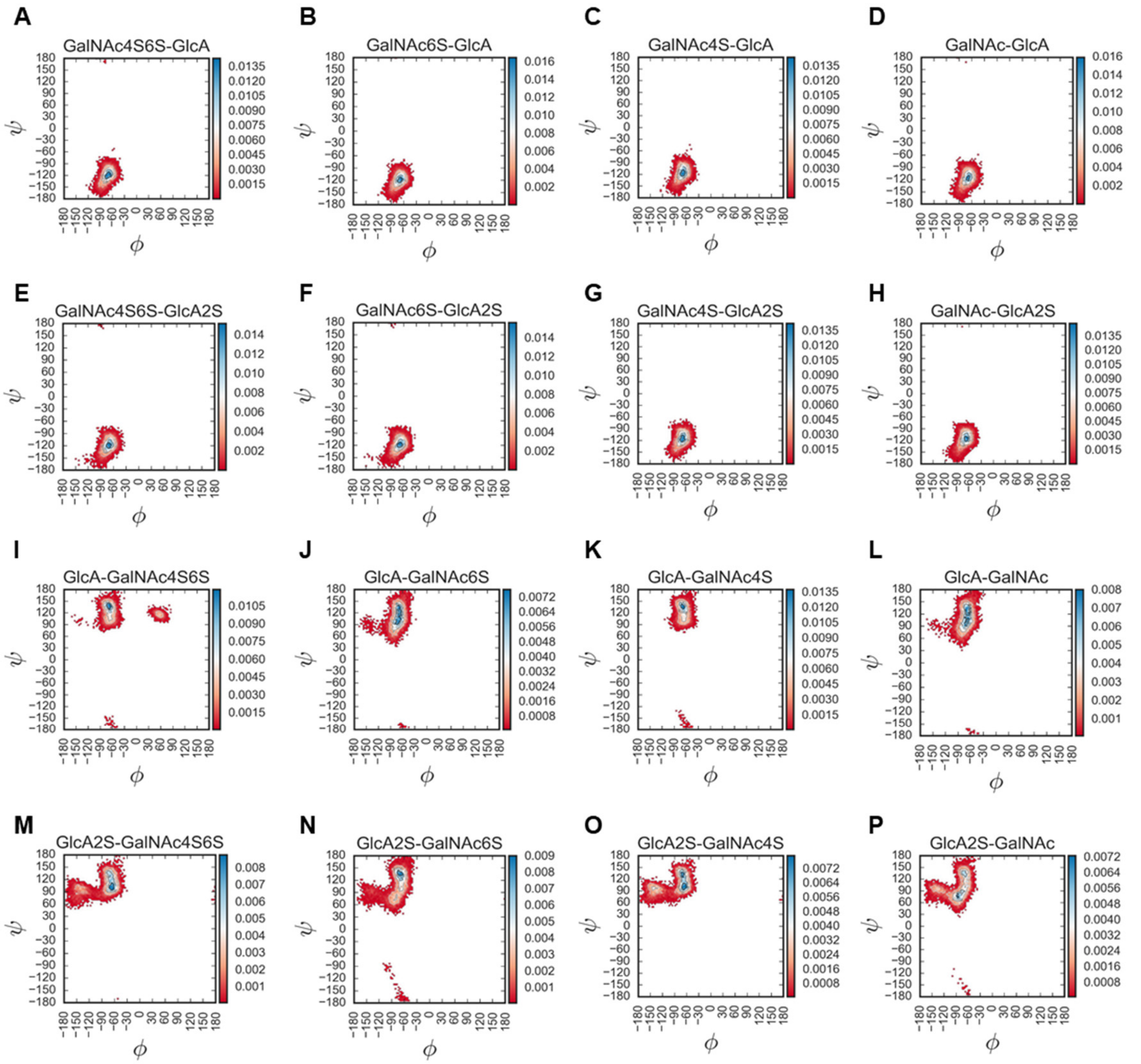

Figure S7. A plot of torsional density distribution as a function of Φ and Ψ for all 16 CS disaccharides. Torsional angles Φ and Ψ are defined in Figure 1. Each frame of the trajectory was analyzed for Φ and Ψ values, which were used to assign each frame to unique bins of 3.6° each (360° divided by 100). The number of structures present in each bin was used to calculate the probability density. Blue regions show the highest probability space, whereas red correspond to that with lowest probability.

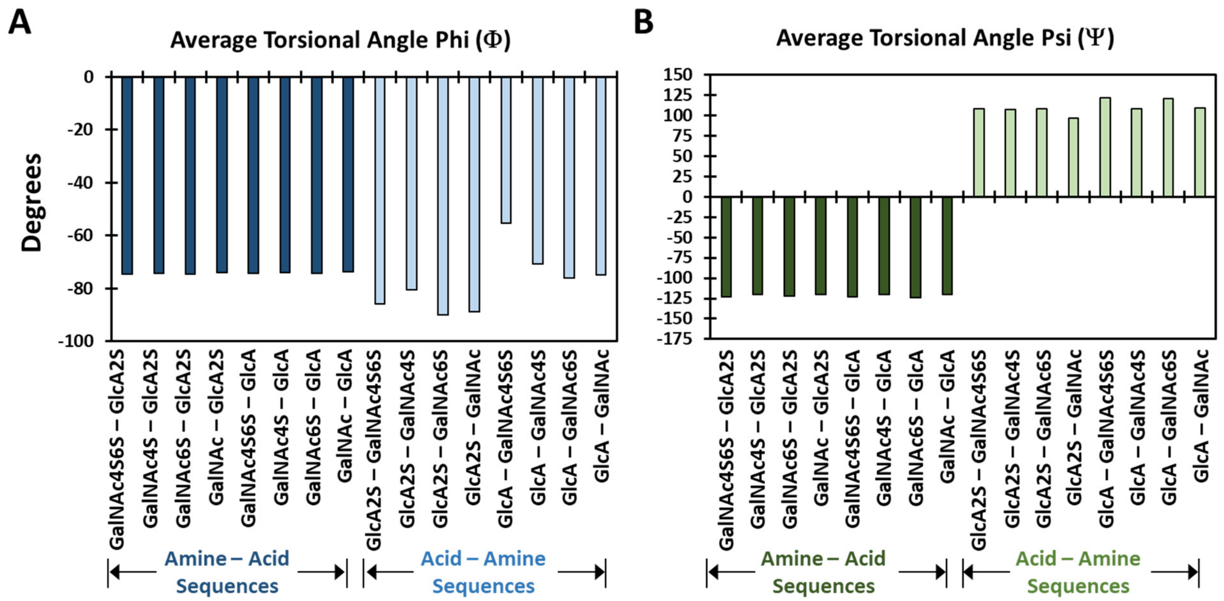

Figure S8. Average torsional angles, phi (A) and psi (B), for each CS disaccharide. Average torsions were obtained from compilation of data corresponding to the blue regions of interest in Figure S6. The definition of phi and psi is provided in the Methods section and Figure 1.

Table S1. Torsional angles calculated from all available experimental structures of CS oligosaccharides from the PDB (www.rcsb.org).

PDB ID	Sequence Length	Sequence	GalNAc—GlcA (1→4-linkage) Φ (O5-C1-O4'-C4')	GalNAc—GlcA (1→4-linkage) Ψ (C1-O4'-C4'-C5')	GlcA—GalNAc (1→3-linkage) Φ (O5-C1-O3'-C3')	GlcA—GalNAc (1→3-linkage) Ψ (C1-O3'-C3'-C4')
**4c4m**	4	(GlcA-GalNAc4S)$_2$	-65.1	-107.3	-56.3	109.0
**2wda**	2	GlcA-GalNAc4S			-81.2	130.0
**2ahg**	2	GlcA-GalNAc			-85.4	99.3
**2kqo**	6	(GlcA-GalNAc)$_3$	-73.3	-116.3	-71.6	108.8
**1c4s**	6	(GalNAc4S-GlcA)$_3$	-79.9	-128.7	-86.4	128.0
**1rwc**	2	GlcA-GalNAc			-62.7	115.0
**1rwf**	4	(GlcA-GalNAc4S)$_2$	-65.6	-148.6	-57.4	129.4
**1rwg**	4	(GlcA-GalNAc4S)$_2$	-69.0	-149.1	-65.5	134.1
**1rwh**	4	(GlcA-GalNAc4S)$_2$	-75.7	-148.0	-67.0	133.2
**1hmw**	4	(GlcA-GalNAc4S)$_2$	-87.5	-141.8	-69.4	94.5
**1ofm**	4	(GlcA-GalNAc4S)$_2$	-69.3	-108.0	-89.2	107.7
**3c9e**	6	(GalNAc4S-GlcA)$_3$	-53.0	-117.7	55.8	122.4
**3h7d**	6	(GalNAc4S-GlcA)$_3$	-95.4	-133.3	-88.8	141.0
**3ank**	2	GlcA-GalNAc6S			-94.2	98.0
**2d8l**	2	GlcA-GalNAc			-72.4	128.1

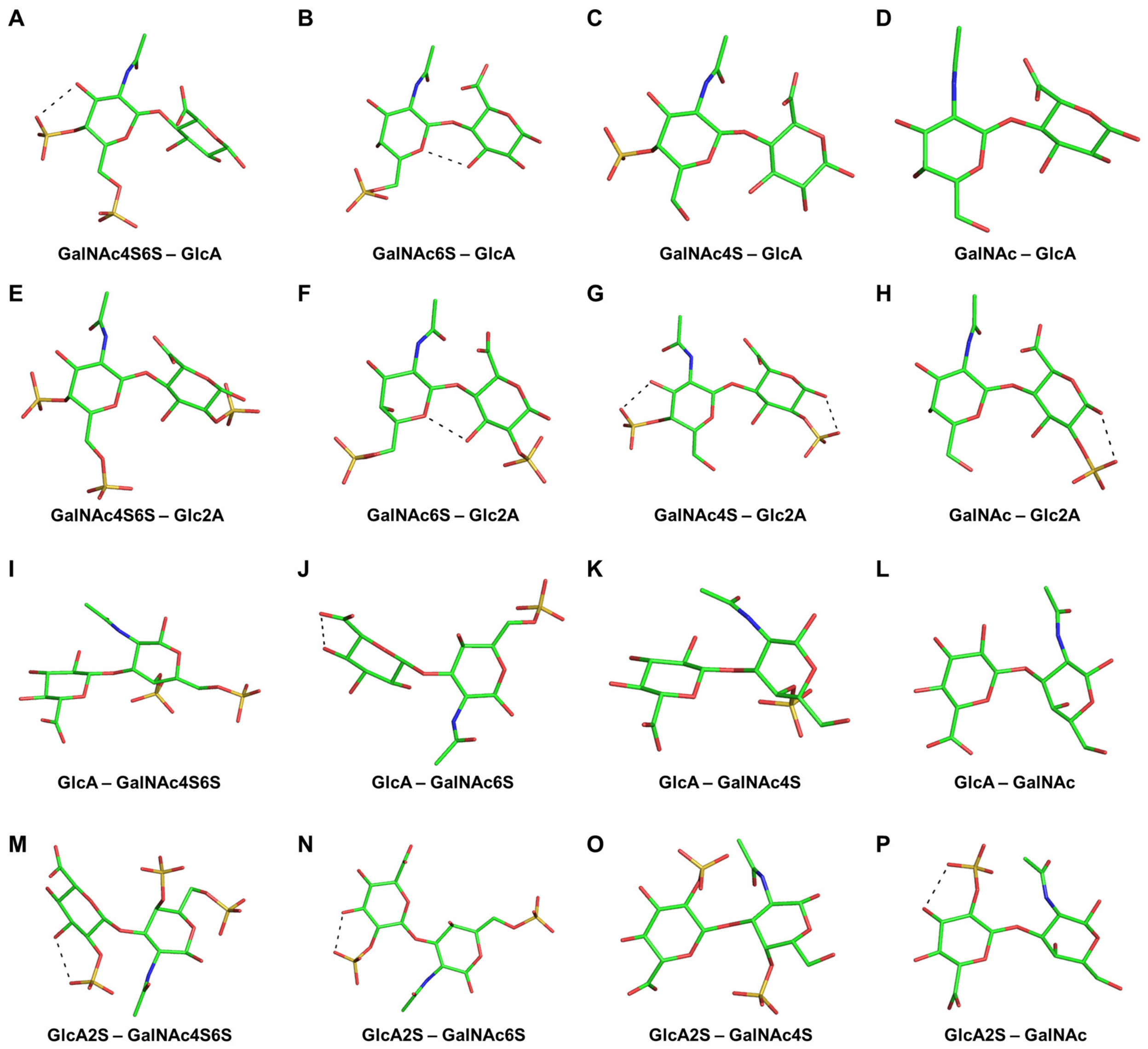

Figure S9. The global energy minimum and its intra-molecular hydrogen bond interactions (shown as dotted lines) for each CS disaccharide.

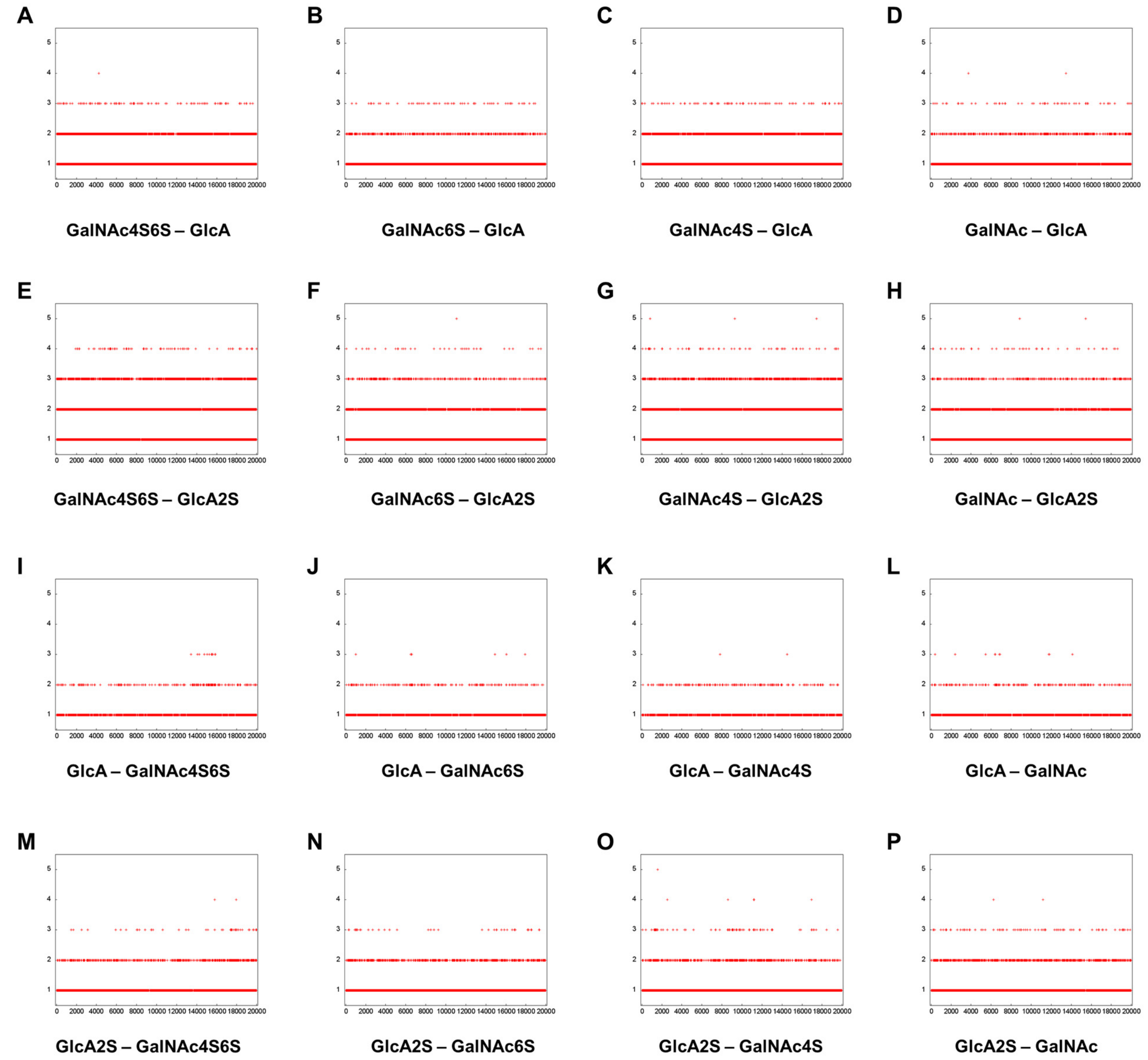

Figure S10. The number of intra-molecular hydrogen bonds formed for each disaccharide as a function of the MD trajectory over 20,000 frames from the start (time 0) to end (time 20 ns).

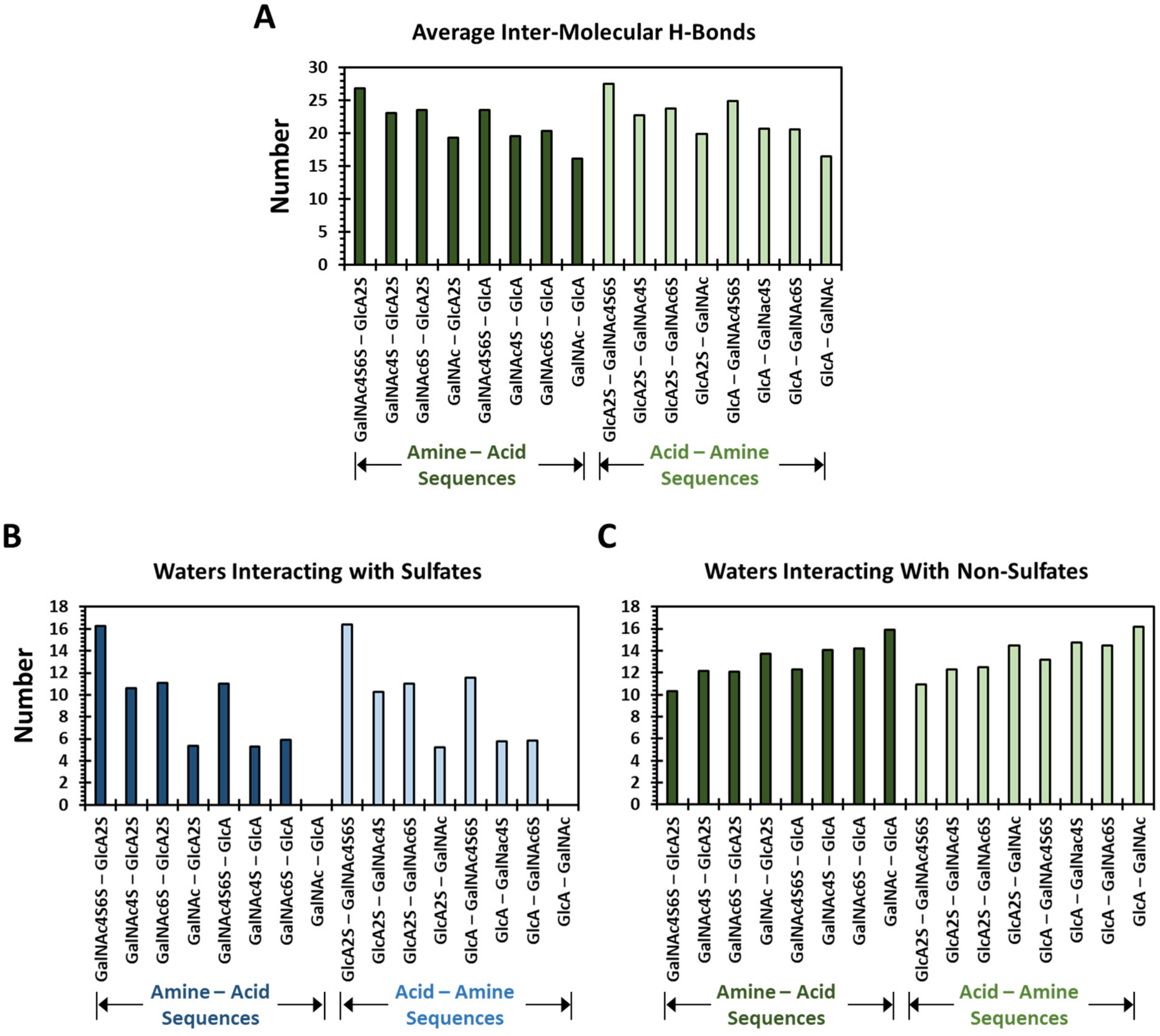

Figure S11. Number of inter-molecular H-bonds observed in MD simulations. A) Average number of inter-molecular H-bonds, i.e., with water molecules, observed for all CS disaccharides over the MD trajectory. B) and C) Number of water molecules H-bonding to either a sulfate (B) or non-sulfate atom (C) of each disaccharide (within a shell of 3 Å) calculated from 20,000 MD frames The criteria for defining interacting water molecules is described in the Methods section.

Table S2. Information on H-bond donor and acceptor pairs for 1→4-linked CS disaccharides from the MD trajectories. Intra-residue interactions are not presented. Also H-bonds noted in less than 0.01 % frames are not presented.

CS Disaccharide	Acceptor	Donor	% Frames
GalNAc4S6S-GlcA2S	GlcA(2S)_2@O6B	GalNAc(4S6S)_1@H2N	1.90
	GlcA(2S)_2@O6A	GalNAc(4S6S)_1@H2N	1.63
	GalNAc(4S6S)_1@O5	GlcA(2S)_2@H3O	46.77
	GalNAc(4S6S)SO3	GlcA(2S)_2@H3O	1.53
	GalNAc(4S6S)SO1	GlcA(2S)_2@H3O	1.49
	GalNAc(4S6S)SO2	GlcA(2S)_2@H3O	1.29
GalNAc6S-GlcA2S	GlcA(2S)_2@O6A	GalNAc(6S)_1@H2N	2.04
	GlcA(2S)_2@O6B	GalNAc(6S)_1@H2N	1.90
	GalNAc(6S)_1@O5	GlcA(2S)_2@H3O	46.33
	GalNAc(6S)SO3	GlcA(2S)_2@H3O	2.54
	GalNAc(6S)SO1	GlcA(2S)_2@H3O	2.23
	GalNAc(6S)SO2	GlcA(2S)_2@H3O	1.55
GalNAc4S-GlcA2S	GlcA(2S)_2@O6B	GalNAc(4S)_1@H2N	2.66
	GlcA(2S)_2@O6A	GalNAc(4S)_1@H2N	1.65
	GlcA(2S)_2@O3	GalNAc(4S)_1@H6O	0.40
	GalNAc(4S)_1@O5	GlcA(2S)_2@H3O	38.35
	GalNAc(4S)_1@O6	GlcA(2S)_2@H3O	0.02
GalNAc-GlcA2S	GlcA(2S)_2@O6A	GalNAc_1@H2N	2.28
	GlcA(2S)_2@O6B	GalNAc_1@H2N	1.99
	GlcA(2S)_2@O3	GalNAc_1@H6O	0.37
	GalNAc_1@O5	GlcA(2S)_2@H3O	39.35
	GalNAc_1@O6	GlcA(2S)_2@H3O	0.03
GalNAc4S6S-GlcA	GlcA_2@O6B	GalNAc(4S6S)_1@H2N	1.74
	GlcA_2@O6A	GalNAc(4S6S)_1@H2N	1.64
	GalNAc(4S6S)_1@O5	GlcA_2@H3O	46.96
	GalNAc(4S6S)_1@O6	GlcA_2@H3O	0.05
	GalNAc(4S6S)SO1	GlcA_2@H3O	1.74
	GalNAc(4S6S)SO3	GlcA_2@H3O	1.52
	GalNAc(4S6S)SO2	GlcA_2@H3O	1.44
	GalNAc(4S6S)SO1	GlcA_2@H2O	0.06
GalNAc6S-GlcA	GlcA_2@O6B	GalNAc(6S)_1@H2N	1.79
	GlcA_2@O6A	GalNAc(6S)_1@H2N	1.50
	GalNAc(6S)_1@O5	GlcA_2@H3O	49.09
	GalNAc(6S)SO1	GlcA_2@H3O	2.32
	GalNAc(6S)SO2	GlcA_2@H3O	2.03
	GalNAc(6S)SO3	GlcA_2@H3O	1.32
GalNAc4S-GlcA	GlcA_2@O6B	GalNAc(4S)_1@H2N	2.62
	GlcA_2@O6A	GalNAc(4S)_1@H2N	1.46
	GlcA_2@O3	GalNAc(4S)_1@H6O	0.29
	GlcA_2@O6B	GalNAc(4S)_1@H6O	0.06
	GalNAc(4S)_1@O5	GlcA_2@H3O	39.11
	GalNAc(4S)_1@O6	GlcA_2@H3O	0.03
GalNAc-GlcA	GlcA_2@O6A	GalNAc_1@H2N	2.05
	GlcA_2@O6B	GalNAc_1@H2N	1.92
	GlcA_2@O3	GalNAc_1@H6O	0.24
	GlcA_2@O6B	GalNAc_1@H6O	0.04
	GlcA_2@O6A	GalNAc_1@H6O	0.03
	GalNAc_1@O5	GlcA_2@H3O	41.86
	GalNAc_1@O6	GlcA_2@H3O	0.02

Table S3. Information on H-bond donor and acceptor pairs for 1→3-linked CS disaccharides from the MD trajectories. Intra-residue interactions are not presented. Also H-bonds noted in less than 0.01 % frames are not presented.

CS Disaccharide	Acceptor	Donor	% Frames
GlcA2S-GalNAc4S6S	GlcA(2S)SO1	GalNAc(4S6S)_2@H2N	1.26
	GlcA(2S)SO2	GalNAc(4S6S)_2@H2N	1.11
	GlcA(2S)SO3	GalNAc(4S6S)_2@H2N	1.00
	GlcA(2S)_1@O2	GalNAc(4S6S)_2@H2N	1.01
GlcA2S-GalNAc6S	GlcA(2S)SO1	GalNAc(6S)_2@H4O	0.81
	GlcA(2S)SO2	GalNAc(6S)_2@H2N	0.56
	GlcA(2S)SO2	GalNAc(6S)_2@H4O	0.54
	GlcA(2S)SO3	GalNAc(6S)_2@H2N	0.44
	GlcA(2S)SO1	GalNAc(6S)_2@H2N	0.41
	GlcA(2S)SO3	GalNAc(6S)_2@H4O	0.22
	GlcA(2S)_1@O5	GalNAc(6S)_2@H4O	2.19
	GlcA(2S)_1@O2	GalNAc(6S)_2@H2N	0.22
	GlcA(2S)_1@O2	GalNAc(6S)_2@H4O	0.15
GlcA2S-GalNAc4S	GlcA(2S)_1@O2	GalNAc(4S)_2@H2N	1.19
	GlcA(2S)SO2	GalNAc(4S)_2@H2N	2.16
	GlcA(2S)SO1	GalNAc(4S)_2@H2N	1.1
	GlcA(2S)SO3	GalNAc(4S)_2@H2N	0.95
GlcA2S-GalNAc	GlcA(2S)_1@O5	GalNAc_2@H4O	4.19
	GlcA(2S)_1@O2	GalNAc_2@H2N	0.59
	GlcA(2S)SO2	GalNAc_2@H2N	1.28
	GlcA(2S)SO1	GalNAc_2@H2N	1.28
	GlcA(2S)SO3	GalNAc_2@H2N	1.12
	GlcA(2S)SO1	GalNAc_2@H4O	0.52
	GlcA(2S)SO3	GalNAc_2@H4O	0.17
	GlcA(2S)SO2	GalNAc_2@H4O	0.08
GlcA-GalNAc4S6S	GlcA_1@O5	GalNAc(4S6S)_2@H2N	0.32
	GlcA_1@O2	GalNAc(4S6S)_2@H2N	0.06
	GalNAc(4S6S)_2@N2	GlcA_1@H2O	0.01
	GalNAc(4S6S)_2@O3	GlcA_1@H2O	0.01
	GalNAc(4S6S)SO1	GlcA_1@H2O	4.59
	GalNAc(4S6S)SO3	GlcA_1@H2O	4.29
	GalNAc(4S6S)SO2	GlcA_1@H2O	2.62
	GalNAc(4S6S)_2@O2N	GlcA_1@H2O	5.16
GlcA-GalNAc6S	GlcA_1@O5	GalNAc(6S)_2@H4O	1.88
	GlcA_1@O2	GalNAc(6S)_2@H2N	0.06
	GlcA_1@O5	GalNAc(6S)_2@H2N	0.01
	GalNAc(6S)_2@O3	GlcA_1@H2O	0.01
	GalNAc(6S)_2@O2N	GlcA_1@H2O	8.48
GlcA-GalNAc4S	GlcA_1@O5	GalNAc(4S)_2@H2N	0.01
	GlcA_1@O2	GalNAc(4S)_2@H2N	0.01
	GalNAc(4S)_2@O3	GlcA_1@H2O	0.01
	GalNAc(4S)_2@O2N	GlcA_1@H2O	6.04
	GalNAc(4S)SO2	GlcA_1@H2O	0.32
	GalNAc(4S)SO1	GlcA_1@H2O	0.18
	GalNAc(4S)SO3	GlcA_1@H2O	0.18
GlcA-GalNAc	GlcA_1@O5	GalNAc_2@H4O	1.65
	GlcA_1@O2	GalNAc_2@H2N	0.01
	GlcA_1@O5	GalNAc_2@H2N	0.01
	GalNAc_2@O2N	GlcA_1@H2O	8.21
	GalNAc_2@O3	GlcA_1@H2O	0.01

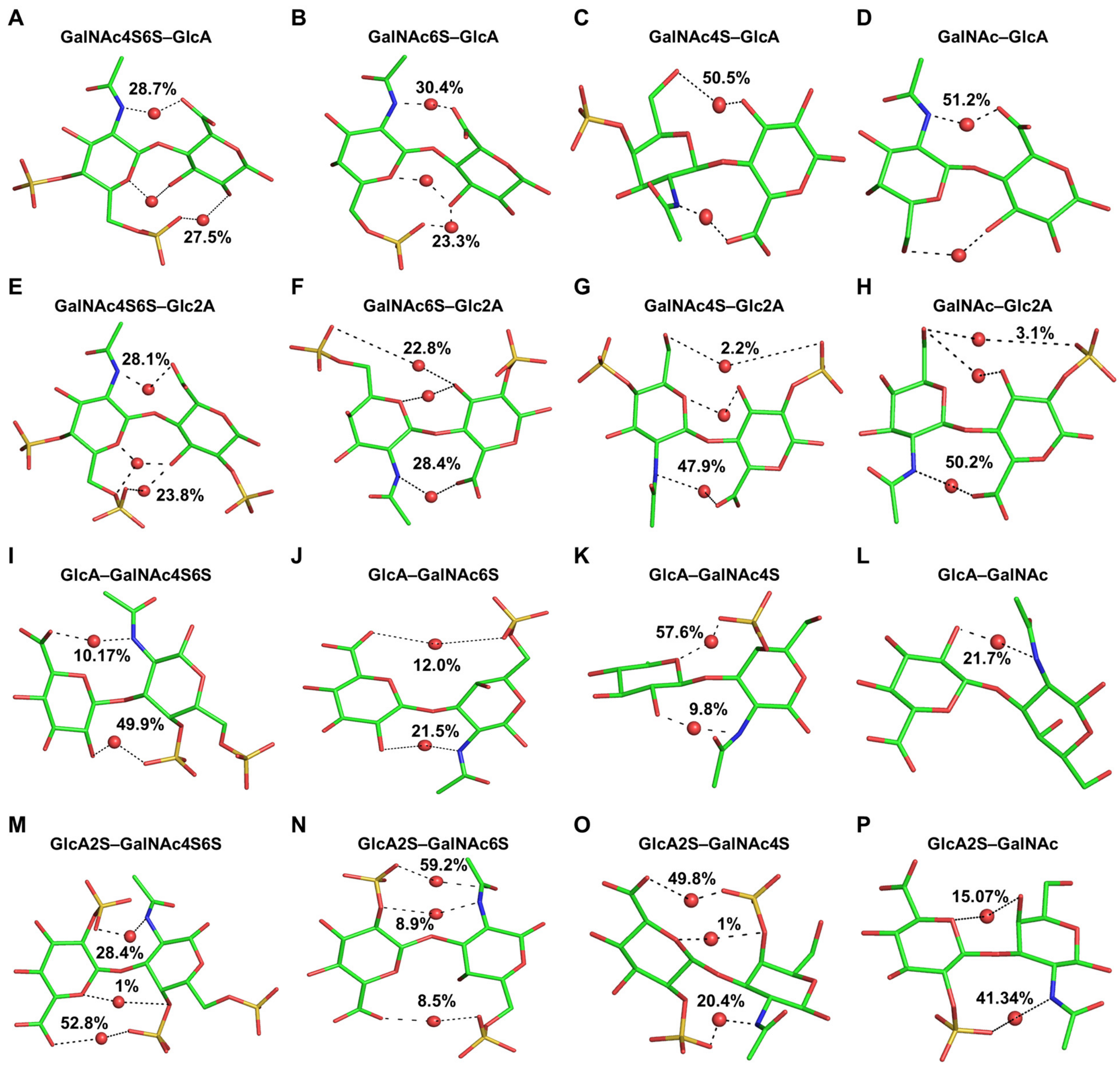

Figure S12. Inter-residue bridging water molecules observed during MD study of all 16 CS disaccharides and their population (in %) from the 20,000 frames of each trajectory. The bridging water is shown as red sphere (oxygen atom) and the interactions are shown as dotted lines with % of occurrence.

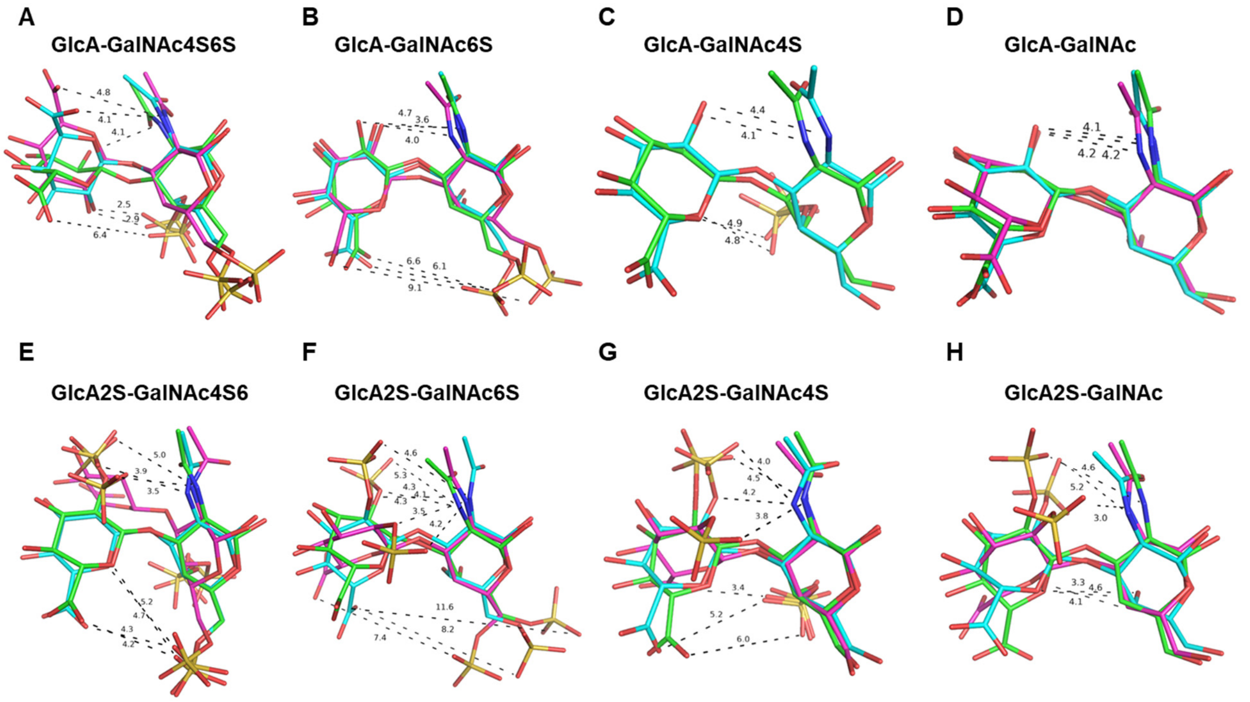

Figure S13. Representative structures from the three different regions of probability density plot (**Figure S6**) and their interactions with water in MD simulations. Structure of overlaid to highlight the differences in water-mediated interactions. The overlaps present differences in Φ and Ψ for 1→3-linked GlcA-GalNAc sequences. The structure with green sticks shows the highest probability region; next in probability order is cyan sticks; and finally, pink sticks show lowest probability. The listed distances are measured between the closest atom pairs of the two residues of the sequences and shown as dotted lines. These lines indicate a possible direct or water-mediated interaction between the two monosaccharides.

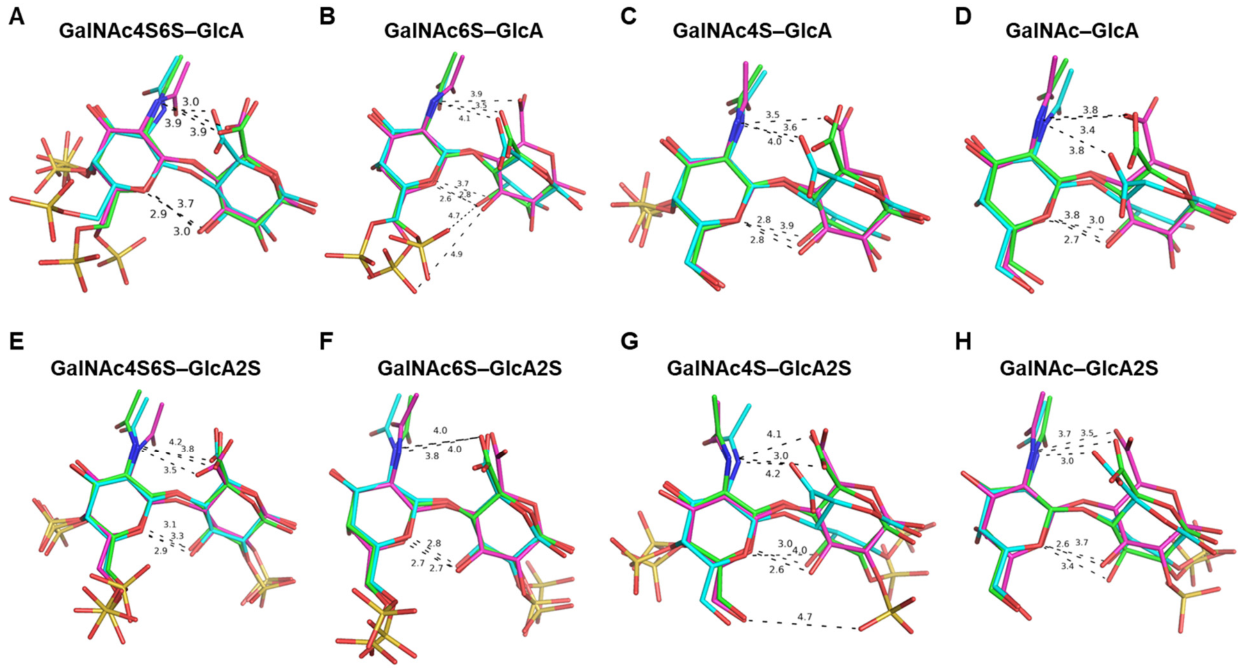

Figure S14. Representative structures from the three different regions of probability density plot (**Figure S6**) and their interactions with water in MD simulations. Structure of overlaid to highlight the differences in water-mediated interactions. The overlaps present differences in Φ and Ψ for 1→4-linked GalNAc-GlcA sequences. The structure with green sticks shows the highest probability region; next in probability order is cyan sticks; and finally, pink sticks show lowest probability. The listed distances are measured between the closest atom pairs of the two residues of the sequences and shown as dotted lines. These lines indicate a possible direct or water-mediated interaction between the two monosaccharides.

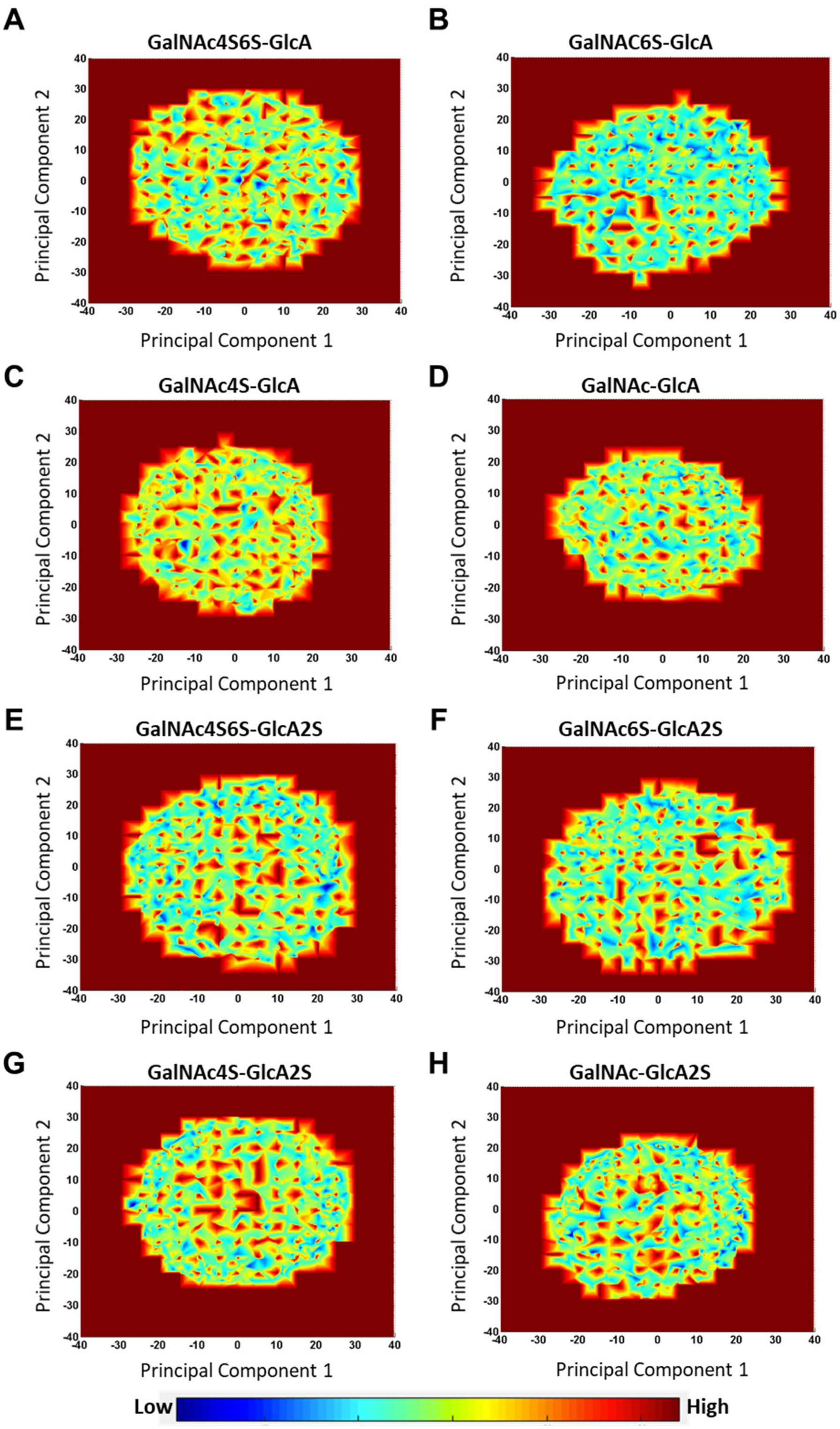

Figure S15. Energy landscape of the eight 1→4-linked disaccharides. The *x*- and *y*-axis are the first two principal components derived from principal component analysis (PCA) of each MD trajectory, while potential energy of each frame is potted along the *z*-axis and projected in the 2D space.

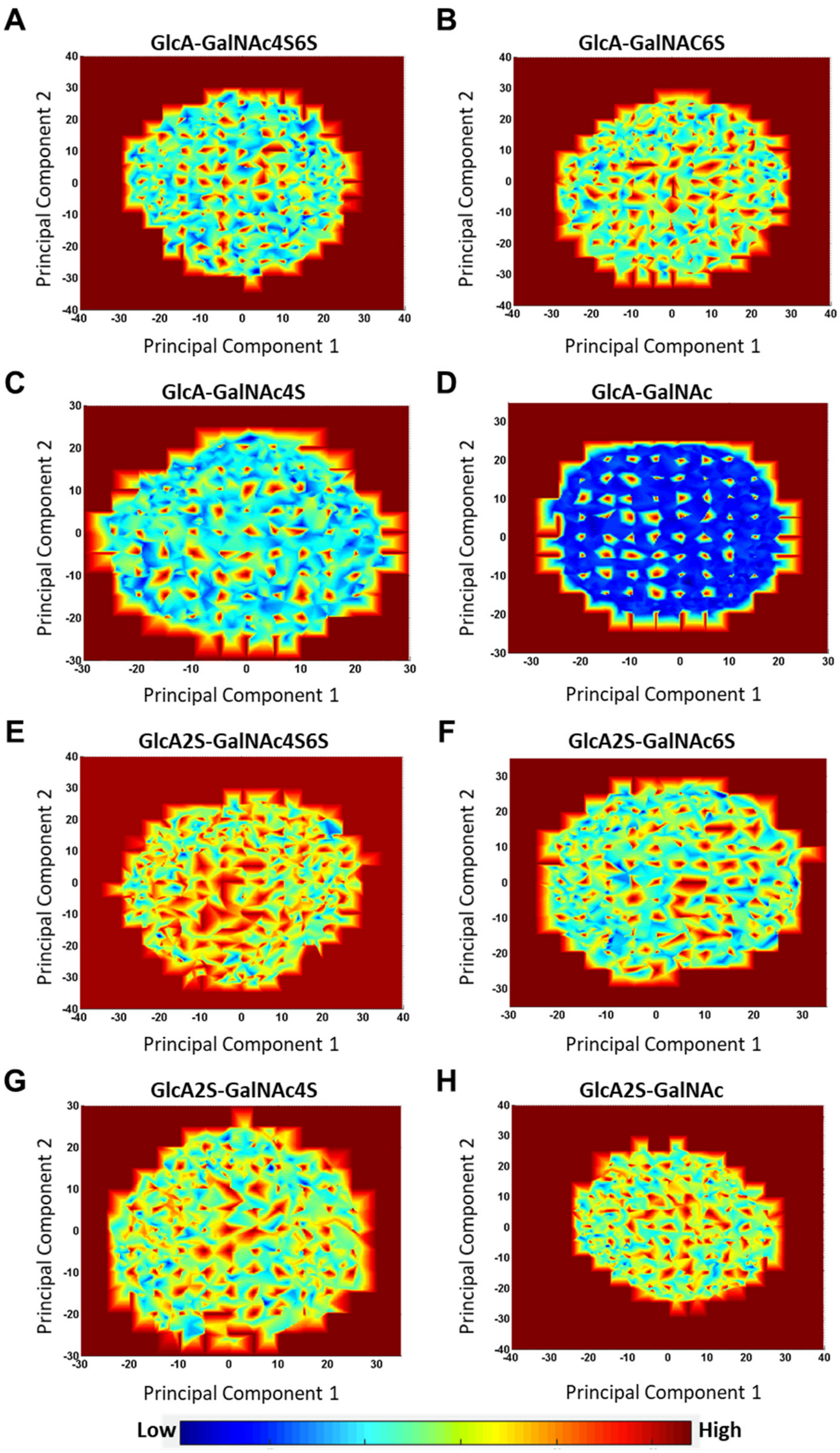

Figure S16. Energy landscape of the eight 1→3-linked disaccharides. The *x*- and *y*-axis are the first two principal components derived from principal component analysis (PCA) of each MD trajectory, while potential energy of each frame is potted along the *z*-axis and projected in the 2D space.

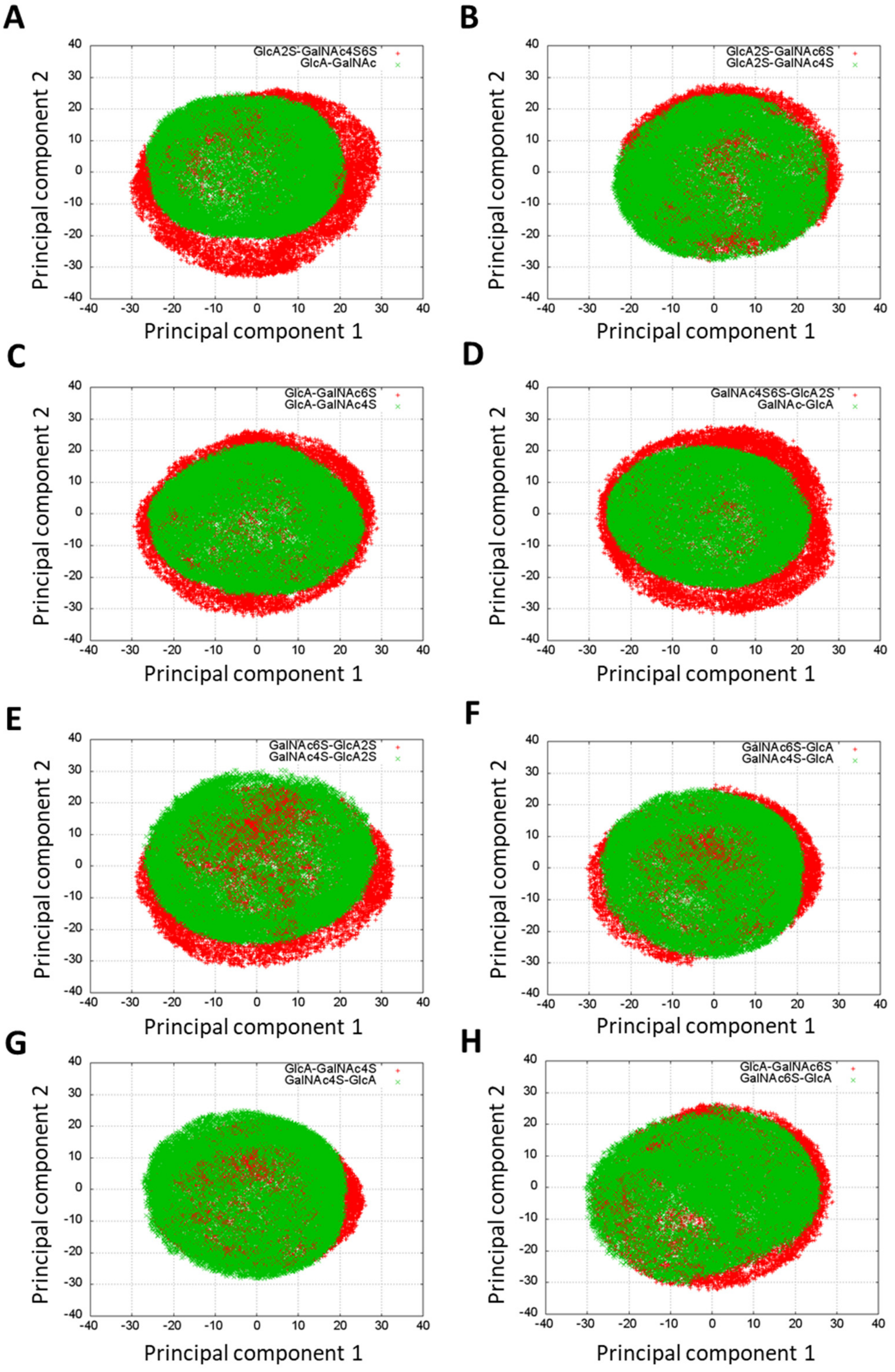

Figure S17. Comparisons of first two principal components derived from principal component analysis of the MD trajectories of 16 CS disaccharides. The reduced subspace clarifies the similarity and dissimilarity of conformational entropy across different disaccharides.
